# Caspase-2 deficiency drives pathogenic liver polyploidy and increases age-associated hepatocellular carcinoma in mice

**DOI:** 10.1126/sciadv.aeb2571

**Published:** 2026-01-01

**Authors:** Loretta Dorstyn, Yoon Lim, Jack Scanlan, Emma McLennan, Dylan De Bellis, Michael Katschner, John Finnie, Samantha Emery-Corbin, Jumana Yousef, Laura F. Dagley, Chung H. Kok, Sonia S. Shah, Chiaki Takahashi, Mark A. Febbraio, Sharad Kumar

**Affiliations:** ^1^ Centre for Cancer Biology, University of South Australia, GPO Box 2471, Adelaide, SA 5001, Australia.; ^2^ College of Health, Adelaide University, Adelaide, SA 5005, Australia.; ^3^ Monash Institute of Pharmaceutical Sciences, Monash University, 399 Royal Parade, Parkville, VIC 3052, Australia.; ^4^ Adelaide Medical School, The University of Adelaide, North Terrace, Adelaide, SA 5005, Australia.; ^5^ Monash Biomedicine Discovery Institute, Monash University, Clayton, VIC 3800, Australia.; ^6^ Walter and Eliza Hall Institute of Medical Research, 1G Royal Parade, Parkville, VIC 3052, Australia.; ^7^ University of Melbourne, Melbourne, VIC 3010, Australia.; ^8^ Department of Genetics and Molecular Pathology, SA Pathology, Adelaide, SA 5000, Australia.; ^9^ Cancer Research Institute, Kanazawa University, Kanazawa, Japan.

## Abstract

Hepatocyte polyploidization promotes liver homeostasis by enhancing resistance to cellular stress. Caspase-2, a proapoptotic protease, restricts polyploidization by deleting polyploid and aneuploid cells. While caspase-2 protects against diet-induced hepatic injury, it also acts as a tumor suppressor by controlling genomic instability and oxidative stress. To investigate these roles, we assessed hepatic ploidy dynamics, liver damage, and age-associated tumorigenesis in caspase-2–deficient and catalytically inactive mutant mice. We found that caspase-2 loss promotes early-onset hepatocyte hyperpolyploidy, accompanied by progressive liver inflammation, fibrosis, oxidative liver damage, ferroptosis, and higher incidence of spontaneous hepatocellular carcinoma in aged animals. Proteomic profiling revealed a pathogenic polyploidy–associated signature associated with caspase-2 deficiency and increased predisposition to liver disease and malignancy. These findings establish caspase-2 enzymatic activity as a critical regulator of hepatic genome stability and preventing age-related liver cancer that strongly argue against therapeutic caspase-2 inhibition as a strategy for managing liver injury or cancer risk.

## Teaser

Caspase-2 inactivation promotes pathogenic hepatocyte polyploidy and age-related liver cancer.

## INTRODUCTION

Ploidy imbalances refer to gain and loss of individual chromosomes (aneuploidy) or whole-genome duplication (or polyploidy) that can drive genomic instability (GIN) and tumorigenesis (*1*). While polyploidy fosters tumor cell adaptation, it is also common to many specialized cells (e.g., hepatocytes, megakaryocytes, and heart muscle) and associated with organogenesis, tissue homeostasis, and repair (*2*). In hepatocytes, cellular polyploidy (binucleate polyploidy) is proposed to buffer against hepatotoxic stressors, age-related senescence, and functional decline (*3*–*5*). However, mononucleate polyploidy is often increased in metabolic dysfunction–associated steatotic liver disease (MASLD) and worse prognosis in hepatocellular carcinoma (HCC) (*4*, *6*). A dynamic balance in hepatic polyploid states is therefore critical for liver homeostasis and tumor suppression, but how this process is controlled during aging is still unclear (*4*).

The apoptotic caspase, caspase-2, has an important function in limiting aneuploidy and polyploidy, as part of its roles in apoptosis, tumor suppression, and aging (*7*–*10*). Mechanistically, caspase-2 is activated following mitotic catastrophe (MC) (*11*, *12*) and triggers the apoptosis pathway via cleavage of the BH3-interacting domain death agonist, BID, to prevent survival of cells with aneuploidy (*13*, *14*). Alternatively, following cytokinesis failure, p53-induced death domain protein 1 (PIDD1)-dependent caspase-2 activation promotes cleavage of mouse double minute 2 protein, MDM2, to drive p53-mediated cell cycle arrest and/or apoptosis of polyploid cells (*12*, *15*). As a consequence, *Casp2* knockout (*Casp2^−/−^*) cells show increased DNA damage and GIN due to inefficient apoptotic killing of aberrant mitotic cells (*14*) during aging or replicative stress (*13*, *16*–*18*). While this feature can promote tumorigenesis in several mouse tumor models in the absence of *caspase-2* (*7*–*10*, *19*–*22*), there are also contradictory reports that increased hepatocyte polyploidy in *Casp2^−/−^* mice fosters protection against carcinogen (diethylnitrosamine)–induced HCC development (*3*, *22*–*24*). This suggests that regulation of liver ploidy dynamics is critical for liver homeostasis. While caspase-2 proteolytic activity is thought to be required to restrict the proliferation and survival of cells with ploidy imbalances (*9*, *25*), this has not been directly tested.

Further to its role in maintaining liver homeostasis, loss of *Casp2* in young adult mice also offers protection against high-fat or high-fructose diet-induced steatotic liver disease (MASLD) and metabolic dysfunction–associated steatohepatitis (MASH) (*26*–*31*). In addition, *Casp2^−/−^* young and old mice exhibit improved fat metabolism and reduced total body fat, partly due to reduced lipogenesis and increased lipolysis following a high-fat diet (*10*, *29*, *32*). Relevantly, increased *CASP2* levels are associated with liver disease and reduced levels with more favorable prognosis for HCC in patients (*29*, *33*). This has led to development of caspase-2 inhibitors as a means to block diet-induced pathogenic progression of fatty liver disease and HCC (*29*, *33*). Caspase-2 loss in mice also impairs antioxidant defense responses (*9*, *25*) and leads to increased age-related oxidative liver damage and altered metabolic regulation (*10*, *32*). As part of the approach to targeting caspase-2, it is imperative to understand its roles in limiting polyploidy and whether this is linked to its role in regulating oxidative stress and metabolism during aging (*10*, *26*, *30*, *32*) to decipher the long-term physiological benefits or effects of blocking caspase-2 function for age-related liver homeostasis.

To investigate and assess the role and requirement of caspase-2 proteolytic function in liver ploidy control during aging homeostasis, we examined the dynamics of liver polyploidy and liver damage during aging in *Casp2^−/−^* and catalytic mutant (*Casp2^C320S^*, Cys320→Ser) mice. Furthermore, we carried out global liver proteomics to define the age-related protein changes associated with liver ploidy status. Our data provide the first critical evidence that loss of caspase-2 or its enzymatic activity is adverse for liver homeostasis by promoting early liver hyperpolyploidy that is associated with a pathogenic polyploidy–associated liver disease (ppaLD) protein signature and increased predisposition to chronic hepatitis–like liver disease, ferroptosis, and HCC. Notably, our findings reveal that caspase-2 roles in tumor suppression and hepatosteatosis control are separable and demonstrate that long-term inhibition of caspase-2 catalytic function disrupts the liver polyploid balance that augments liver pathophysiology and tumorigenesis.

## RESULTS

### Liver hyperpolyploidy is increased in young *Casp2^C320S^* mice

**Fig. 1.**
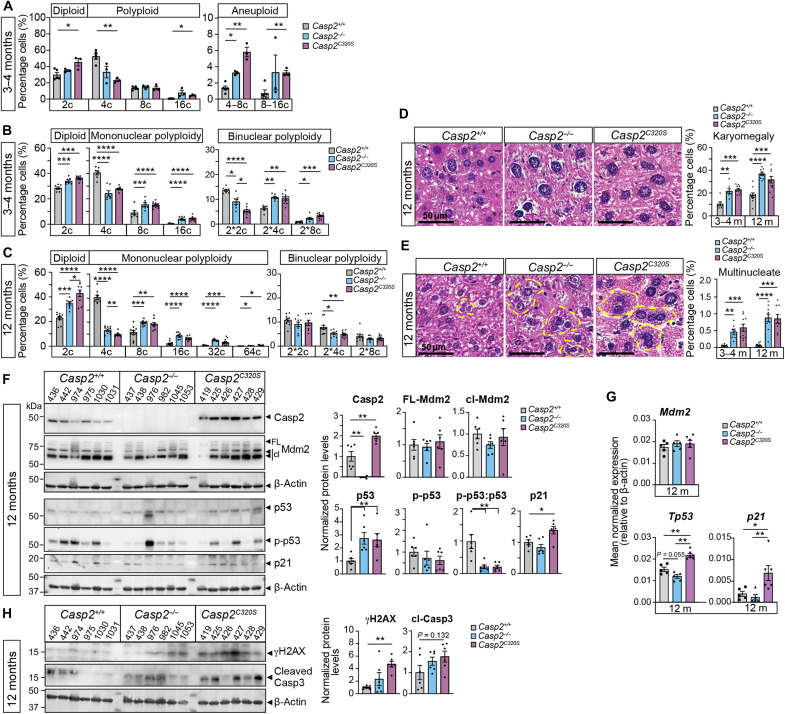
Hepatocyte hyperpolyploidy and aneuploidy in caspase-2-deficient mice. (**A**) Quantitation of the DNA content in hepatocytes from 3- to 4–month (m)–old *Casp2^+/+^*, *Casp2^−/−^*, and *Casp2^C320S^* mice by flow cytometry. The percentage of diploid (2c), polyploid cells (4c, 8c, and 16c), and aneuploid cells (between 4 to 8c and 8 to 16c) are calculated using CytExpert software. Values are means ± SEM; *n* = 3 to 5. *P < 0.05; **P < 0.01. (**B** and **C**) Quantitation of mononuclear and binuclear ploidy from H&E-stained liver sections at 3 months of age (B) and 12 months of age (C), as determined by the area of the spherical nucleus, proportionate to DNA content (2c to 64c) (*37*). Representative images of H&E-stained liver sections from 12-month-old *Casp2^+/+^*, *Casp2^−/−^*, and *Casp2^C320S^* mice, showing (**D**) karyomegalic nuclear morphology of hepatocytes and (**E**) multinucleated cells. Scale bars, 50 μm. In (B) to (E), percentage cells were determined from 10 fields of view (FOVs; ×40 magnification) per liver section. Values are means ± SEM; *n* = 5 mice per genotype. *P < 0.05; **P < 0.01; ***P < 0.001; ****P < 0.0001. (**F**) Representative immunoblots of liver lysates from 12-month-old mice, showing MDM2 cleavage (cl) and levels of p53 and p21, with protein expression levels quantitated as fold change from expression in *Casp2^+/+^* liver lysates, relative to β-actin expression. FL, full length. *n* = 6 independent liver samples per genotype are shown. Significance is indicated as *P < 0.05 and **P < 0.01. (**G**) Quantitative polymerase chain reaction (PCR) analysis of *Mdm2*, *Trp53*, and *p21* expression in 12-month-old livers of the indicated genotypes (*n* = 5 to 6 per genotype). Values are means ± SEM. Significance is indicated as *P < 0.05 and **P < 0.01. (**H**) Representative immunoblots of liver lysates from 12-month-old mice, showing cleaved caspase-3 (Casp3) and γH2AX, with β-actin levels indicating protein loading. Protein expression levels quantitated, as above, from *n* = 6 independent liver samples per genotype, with **P < 0.01.

*Casp2^−/−^* mice not only have increased hepatocyte polyploidy (*12*) but also show differential effects on liver protection and tumor promotion in mouse models of HCC development (*22*, *24*, *34*). While caspase-2-dependent proteolytic cleavage of its substrates (i.e., Bid and Mdm2) mediate its function in MC and ploidy control, the importance of caspase-2 enzymatic function in controlling hepatocyte ploidy has not been directly investigated. To test the importance of caspase-2 proteolytic function in regulating hepatocyte polyploidy in vivo, we carried out DNA content analysis by flow cytometry on freshly isolated hepatocytes from 3- to 4-month-old, enzymatically inactive *Casp2^C320S^* mice. *Casp2^C320S^* mice were previously generated by CRISPR-Cas9–mediated Cys-Ser mutation in the active site (*14*), a mutation that impairs caspase-2 proteolytic activity without affecting protein expression or CARD-mediated dimerization (*35*, *36*). Consistent with observations in *Casp2^−/−^* hepatocytes, we also observed significant changes in hepatocyte ploidy content in *Casp2^C320S^* livers, when compared with wild-type (WT) hepatocytes at this age (Fig. 1A). These changes were marked by an increased number of diploid (2c) and a decreased number of tetraploid hepatocytes (4c), with a concomitant increase in hepatocytes with damaging high-grade polyploidy (hyperpolyploidy) or karyomegaly (16c) in *Casp2^C320S^* livers (Fig. 1A). We also detected an increase in the number of aneuploid hepatocytes (having DNA content between 4c-8c and 8c-16c), demonstrating that caspase-2 catalytic activity is responsible for controlling hepatocyte hyperpolyploidy and ploidy reduction in young mice.

To further distinguish between hepatocytes with binucleate polyploidy and with pathogenic mononucleate polyploidy, we quantitated hepatocyte nuclear size from hematoxylin and eosin (H&E)–stained liver sections based on established methods (*37*) (fig. S1A). These findings were mostly consistent with our DNA content analysis above and demonstrated that the number of mononuclear and binuclear tetraploid cells (i.e., 4c and 2*2c) are reduced in young caspase-2-deficient hepatocytes, which is associated with increases in the diploid (2c) and high-grade polyploid (8c and 16c) cell populations (Fig. 1B). The number of diploid and mononucleated polyploid cells is comparably increased in both *Casp2^−/−^* and *Casp2^C320S^* hepatocytes, whereas the reduction in binucleated 2*2c ploidy in *Casp2^C320S^* is significant compared to both *Casp2^+/+^* and *Casp2^−/−^* hepatocytes (Fig. 1B). Increased ploidy reduction and pathogenic polyploidy are evident at 12 months of age with caspase-2-deficient livers having greater numbers of diploid cells and increased mononucleate polyploidy (8, 16, 32c, and 64c), compared with livers from WT mice (Fig. 1C). This demonstrates an accumulation of pathogenic ploidy in hepatocytes caused by caspase-2 loss, which worsens with aging. These ploidy alterations caused by caspase-2 loss were associated with increased number of hepatocytes with karyomegaly (Fig. 1D), atypical mitotic figures, and nuclear inclusions (membrane-bound, entrapped, and nuclear membrane invagination) (fig. S1, B and C). Notably, the presence of atypical mitotic cells is also further augmented in 12-month-old *Casp2^C320S^* hepatocytes, suggesting that caspase-2 enzymatic mutant has increased mitotic dysfunction with aging. We also observed a greater number of multinucleated hepatocytes, which were mostly trinucleated (with occasional cells having four to five nuclei of heterogeneous sizes), further indicative of impaired cytokinesis in binucleated hepatocytes from caspase-2-deficient livers (Fig. 1E). Collectively, these findings underscore the critical role of caspase-2 activity in preventing the accumulation of mitotically aberrant, hyperpolyploid, and aneuploid hepatocytes with aging.

Caspase-2 activation and function in MC is required to limit hepatocyte polyploidy and is associated with MDM2 cleavage and stabilization of p53 (*13*). Therefore, we next assessed MDM2 and p53 protein levels in liver lysates from young (3- to 4-month-old) and older (12-month-old) *Casp2^−/−^* and *Casp2^C320S^* mice. MDM2 cleavage is observed in livers from all genotypes, with reduced levels observed only in 3-month-old *Casp2^C320S^* mouse livers. However, there are no significant differences in the levels of full-length MDM2 across genotypes at each age (Fig. 1F and fig. S1D), together suggesting caspase-2-independent MDM2 cleavage in the mouse liver. *Casp2^−/−^* and *Casp2^C320S^* livers had higher levels of total p53 compared to caspase-2 WT samples at 12 months of age, with associated increase in p21 protein levels in *Casp2^C320S^* livers (Fig. 1F and fig. S1D). While phosphorylation of p53 (Ser^15^) is detected in multiple samples across genotypes, levels are highly variable, and the phospho-p53/p53 ratio is reduced in both *Casp2^−/−^* and *Casp2^C320S^* hepatocytes, suggesting differential p53 activation dynamics (Fig. 1F). Notably, increased *Trp53* transactivation, together with higher *p21* transcript, was, however, evident in *Casp2^C320S^* (but not *Casp2^−/−^*) livers at 12 months, while levels of *Mdm2* transcript were unchanged across genotypes (Fig. 1G). These findings are suggestive of an enhanced p53-mediated damage response in *Casp2^C320S^* mice. Consistent with this, phosphorylated histone H2AX (γH2AX) levels were also higher in livers from 12-month-old *Casp2^C320S^* mice. Although there is a trend for higher levels of cleaved caspase-3 in caspase-2-deficient livers, this is also variable across liver samples and not significant when compared to *Casp2^+/+^* samples (Fig. 1G), indicating that apoptosis activation is similar in the absence of caspase-2 despite the increased DNA damage. Levels of γH2AX and cleaved caspase-3 were also not altered in young (3- to 4-month-old) livers (fig. S1E), despite the increase in hepatocyte hyperpolyploidy and aneuploidy observed at this age. These data are also consistent with activation of an age-related and p53-mediated DNA damage response in livers from caspase-2-deficient mice.

### Reduced lifespan and premature aging traits in *Casp2^C320S^* mice

**Fig. 2.**
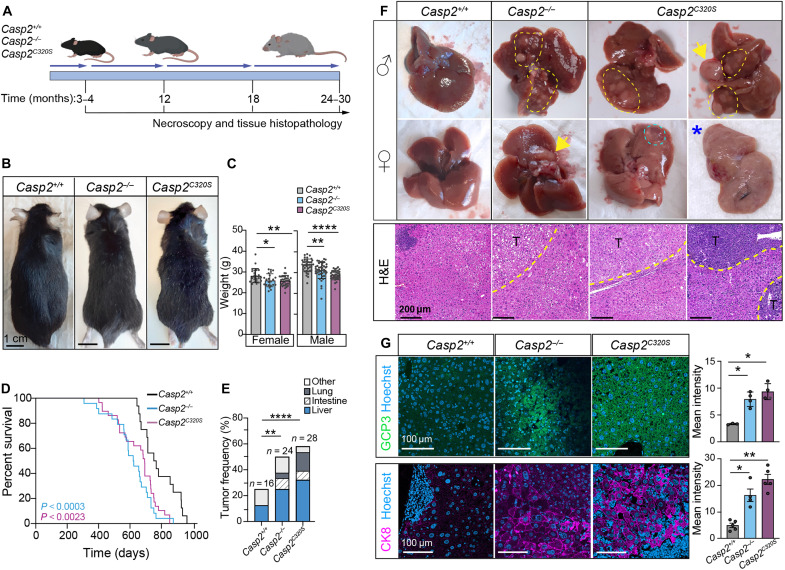
Reduced lifespan in caspase-2-deficient mice is associated with spontaneous age-related liver tumors. (**A**) Experimental design. Cohorts of mice of the indicated genotypes were taken at 3 to 4 months, 12 months, and 18 months of age or at the end of their lifespan (24 to 30 months). (**B**) Representative images of 12-month-old mice showing ruffled fur and mild graying in *Casp2^−/−^* and *Casp2^C320S^* mice. (**C**) Quantitation of total body weights from 10- to 12-month-old female and male mice. Values are means ± SEM; *n* = 22 to 29 female and *n* = 30 to 40 male mice. (**D**) Survival data for *Casp2^+/+^*, *Casp2^−/−^*, and *Casp2^C320S^* mice. Data analyzed using Fisher’s exact test, with significance levels indicated (*Casp2^+/+^*, *n* = 16; *Casp2^−/−^*, *n* = 24; *Casp2^C320S^*, *n* = 28). (**E**) Tumor frequency in *Casp2^+/+^*, *Casp2^−/−^*, and *Casp2^C320S^* mice, as observed by the presence of macroscopic nodules/masses on the indicated tissues. Statistical analysis performed using χ^2^ test. Significance is indicated as *P < 0.05, **P < 0.01, and ****P < 0.0001. (**F**) Representative images of liver architecture and H&E-stained liver sections from old *Casp2^+/+^*, *Casp2^−/−^*, and *Casp2^C320S^* male and female mice (18 to 28 months). Tumor nodes are indicated with yellow arrows or yellow dashed areas. Liver cyst is bordered by a cyan dashed circle, and asterisk (*) denotes fatty liver. H&E-stained liver sections indicate tumor areas (T) that are outlined. Scale bars, 200 μm. (**G**) Immunofluorescent staining for glypican 3 (GCP3; green) as a marker of HCC and cytokeratin 8 (CK8; magenta), both costained with Hoechst DNA stain (blue), showing positive staining in old *Casp2^−/−^* and *Casp2^C320S^* livers with tumors. Scale bars, 100 μm. Quantitation represents mean fluorescence intensity (in pixels) per FOV across five FOVs per sample. Values are means ± SEM. Significance is indicated as *P < 0.05 and **P < 0.01.

Previous studies have demonstrated that homozygous deletion of *Casp2* (*Casp2^−/−^*) is associated with early-onset aging in mice (*8*, *9*). To examine whether the enhanced liver polyploidy alterations in *Casp2^C320S^* mice further affect aging, we aged cohorts of *Casp2^+/+^*, *Casp2^−/−^*, and *Casp2^C320S^* mice over 18 to 30 months (Fig. 2A), until mice displayed two or more clinical features of distress and unwellness, including hunching, ruffled fur, breathing difficulties, impaired movement, ≥10% weight loss, or palpable tumors.

Consistent with our aging observations in *Casp2^−/−^* mice (*9*), the *Casp2^C320S^* mice also exhibited some early aging phenotypes compared with WT mice (*Casp2^+/+^*), including increased hair ruffling and mild graying in 12-month-old male mice (Fig. 2B and fig. S2A) and reduced body weight in mature adult male and female mice (Fig. 2C). Consistent with previous findings that caspase-2 loss improves fat metabolism (*26*, *32*), aged caspase-2-deficient mice also exhibited reduced total plasma cholesterol, whereas blood glucose levels were unaltered compared to WT mice (fig. S2B). In addition, aged *Casp2^C320S^* mice had smaller adipocytes in gonadal white adipose tissue (gWAT), consistent with the reported phenotype in old *Casp2^−/−^* mice and reduced total body weight (fig. S2C). We examined dermal adipose tissue and quantitated skin layers from old mice (epidermis, dermis, panniculus carnosus, and hypodermis) and demonstrated sex-specific differences in dermal white adipose tissue (dWAT) and skin thinning, including atrophy of the epidermis, muscle, and dWAT in male *Casp2^−/−^* and *Casp2^C320S^* mice (fig. S3, A and B). In females, reduced epidermal and dWAT thickness are only significant in *Casp2^−/−^* mice compared to female *Casp2^+/+^* mice, highlighting sex-dependent alterations in skin thinning and adipose tissue deposits in *Casp2^C320S^* mice. There is some evidence of increased number of enlarged and/or multinucleated skin epidermal cells, characteristic of polyploid cells (fig. S3A), but this is not associated with age-related skin lesions in caspase-2-deficient mice and thus not further analyzed.

Analysis of the mouse survival curves showed that *Casp2^C320S^* mice had a reduced median lifespan (694 days compared with 759 days for *Casp2^+/+^* mice; *P* = 0.048) and a reduced maximal lifespan (853 days compared to 953 days for *Casp2^+/+^* mice; *P* = 0.024), which was similar to that observed in *Casp2^−/−^* mice (Fig. 2D) and consistent with previous findings (*9*). Critically, these findings demonstrate that caspase-2 enzymatic activity is required for normal lifespan and aging homeostasis in mice.

### *Casp2^C320S^* mice develop spontaneous age-related liver tumors

Necroscopy analysis of aged *Casp2^C320S^* mice (15 to 30 months old) found tumor nodules on several tissues including liver, lung, and intestine (both small intestine and colon). *Casp2^C320S^* mice showed increased incidence of these spontaneous age-related tumors (57% mice) compared with *Casp2^+/+^* mice (28.6%), with a prevalence of liver tumor nodules [39% in *Casp2^C320S^* mice compared with 14% in *Casp2^+/+^* mice, *X*^2^ (1, *N* = 14,28) = 16.04, *P* < 0.001] (Fig. 2E). Tumor nodules found in *Casp2^C320S^* lung (7% mice) and intestine (7% mice) were also observed in some *Casp2^−/−^* mice but not in WT *Casp2^+/+^* mice (Fig. 2E). Macroscopic examination identified other common age-related tumors at low incidence in both WT and caspase-2-deficient mice, including abnormal sarcoma-like lumps (nonspecified on limbs), thymomas, lymphomas, and ovarian tumors; however, these were not further characterized histologically. We did not observe enhanced incidence of splenomegaly in aged *Casp2^−/−^* or *Casp2^C320S^* mice (fig. S2D).

Liver masses were observed in both male and female *Casp2^−/−^* and *Casp2^C320S^* mice, variably presenting from 15 to 30 months of age (Fig. 2F). The macroscopic appearance varied from large nodules, especially in *Casp2^C320S^* mice, to multiple smaller nodules, cystic growths, or a mottled zonal pattern of the liver parenchyma, the latter corresponding histologically to periportal and lobular infiltrates and/or fatty changes (steatosis) (Fig. 2F). Notably, the liver–body weight ratio is greater in old caspase-2-deficient mice, consistent with enlarged livers in these mice (fig. S2E). The liver tumor masses were histologically characterized as having foci of cellular alteration (FCA), hepatocellular adenoma (HCA), HCC, or B cell lymphoma (Table 1), with appearance of HCA and HCC more common in *Casp2^−/−^* and *Casp2^C320S^* mice. Microscopically, the tumors showed heterogeneous morphology (Fig. 2F) that ranged from well to poorly differentiated types, some with steatosis and occasional hepatoproliferative lesion, found in the form of an FCA (clear cell type). Several mice did not present with obvious macroscopic liver tumor nodules but are histologically characterized as having B cell lymphoma (Table 1), sometimes associated with metastatic lymphomas and associated splenomegaly. However, while liver lymphoma incidence is similar across genotypes, livers from *Casp2^C320S^* mice showed more severe lymphocytic infiltration and spread throughout the liver tissue in older mice (fig. S2F).

**Table 1.** Caspase-2 deficiency promotes HCC development in mice. Liver tumors were classified as either FCA, HCA, HCC, or lymphoma. Values are represented as number of mice with tumors/total number per group (*n* = 16 for *Casp2^+/+^*, *n* = 24 for *Casp2^−/−^*, and *n* = 28 for *Casp2^C320S^*). Total liver tumor incidence (%) and combined FCA, HCA, and HCC incidence (%) are indicated. Significance is determined by chi-square test: for liver carcinoma incidence, [*Casp2^+/+^* versus *Casp2^C320S^*: χ^2^(1, *N* = 44) = 10.351, **P = 0.0013]. For total tumor incidence, [*Casp2^+/+^* versus *Casp2^−/−^*: χ^2^(1, *N* = 40) = 3.916, *P = 0.048] and [*Casp2^+/+^* versus *Casp2^C320S^*: χ^2^(1, *N* = 44) = 13.333, ***P = 0.0003].

Type of liver neoplasia	*Casp2^+/+^*		*Casp2^−/−^*		*Casp2^C320S^*	
FCA HCA HCC	0/16 1/16 1/16	12.5%	0/24 1/24 4/24	20.8%	1/28 3/28 5/28	32.1%
Lymphoma	2/16	12.5%	4/24	16.7%	5/28	17.8%
Total	4/16	25.0%	9/24	37.5%	14/28	50.0%

Immunohistochemical staining for the HCC marker, glypican 3 (GCP3) in livers harboring tumors, demonstrated positive staining in five of nine *Casp2^C320S^* and two of four *Casp2^−/−^* mice, while the two WT livers with tumor nodules were negative for GCP3, suggesting that these liver tumors were not HCC (Fig. 2G). Overexpression of cytokeratin 8, a surrogate marker for tumor cells, is also detected in almost all hepatocytes from *Casp2^C320S^* and *Casp2^−/−^* mice (Fig. 2G). These data demonstrate that caspase-2 loss and enzymatic inactivation increase the incidence of age-related tumor incidence in the liver, characteristic of HCC.

### *Casp2^C320S^* mice exhibit increased oxidative liver damage and ferroptosis

In addition to accelerated aging and tumorigenesis, pathogenic polyploidy is commonly associated with oxidative injury and can exacerbate age-related liver damage (*2*). Moreover, persistent oxidative stress can augment pathogenic hepatocyte polyploidy, GIN, inflammation, and fibrosis, thereby exacerbating age-related liver injury (*4*). We previously demonstrated that aged *Casp2^−/−^* mice (18 to 24 months old) have increased levels of oxidative liver damage (*9*), including damage to lipids, DNA, and proteins (*22*, *25*). To examine whether the observed increase in hepatocyte hyperpolyploidy and liver tumors is associated with oxidative liver damage in *Casp2^C320S^* mice, we assessed and compared liver histology and circulating liver damage biomarkers at 12 months compared to 18- to 24-month-old tumor-free mice (designated >18 months old). We analyzed samples from male mice to avoid any sex differences that are known to influence age-related liver damage and inflammation, as well as differences in progression of liver disease and tumor onset (*32*, *38*–*41*).

**Fig. 3.**
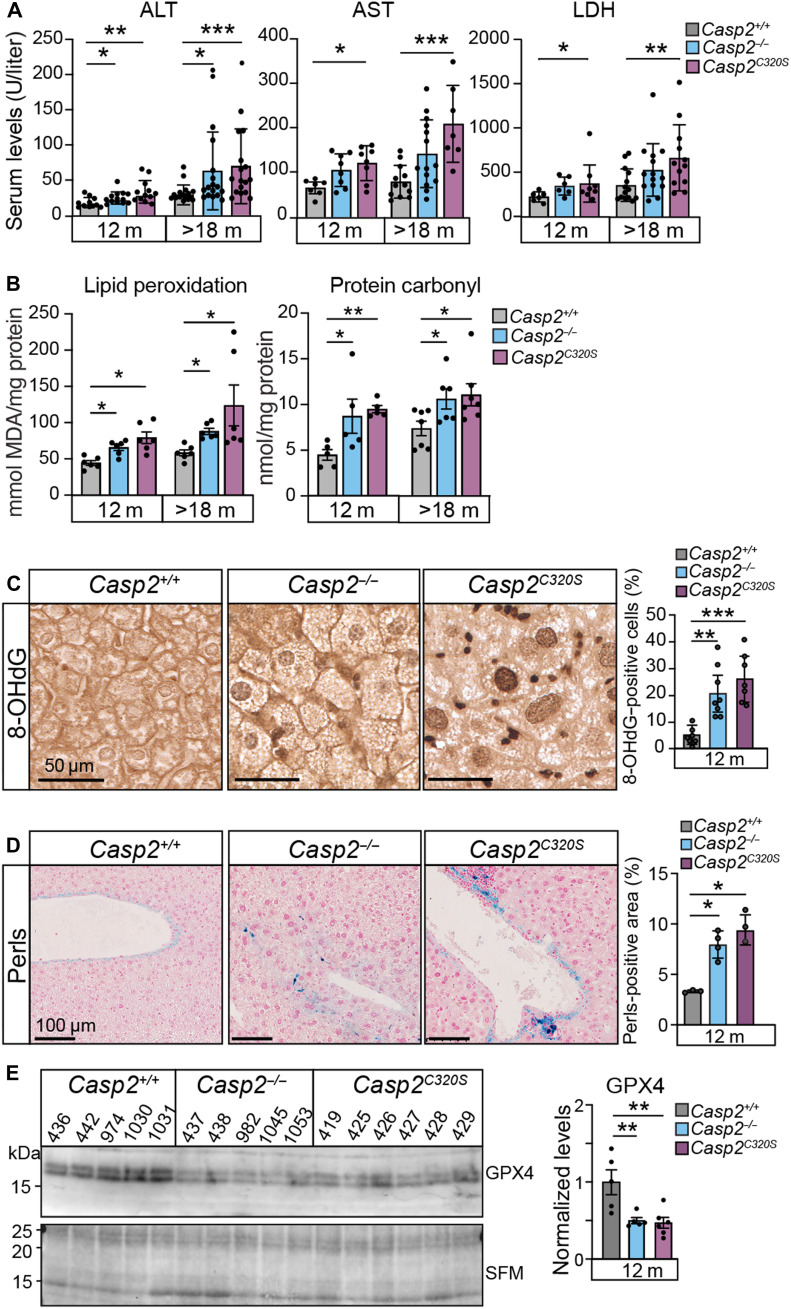
Liver damage and ferroptosis is augmented in *Casp2^C320S^* mice. (**A**) Measurement of plasma markers for liver damage from *Casp2^+/+^*, *Casp2^−/−^*, and *Casp2^C320S^* mice, including ALT, AST, and LDH. Values are means ± SEM. *P < 0.05; **P < 0.01; ***P < 0.001. (**B**) Oxidative damage determined by lipid peroxidation and protein carbonylation in mouse liver lysates from the indicated age cohorts. Values are means ± SEM. *P < 0.05; **P < 0.01. (**C**) Representative images of liver sections stained with 8-OHdG showing increased DNA damage in caspase-2-deficient livers. Scale bars, 50 μm. Quantitation of cells stained positive for 8-OHdG is determined from counting approximately 250 cells across five FOVs per sample, *n* = 5 to 6 livers (independent mice per genotype). Significance is indicated as **P < 0.01 and ***P < 0.001. (**D**) Representative images of liver sections stained with Perls Prussian blue stain showing increased hepatic iron deposition in livers from *Casp2^C320S^* mice. Scale bars, 100 μm. Quantitation of areas stained positive for Perls is shown, determined from at least five FOVs per sample. Significance is indicated as *P < 0.05. (**E**) Immunoblot showing reduced GPX4 levels in liver lysates from *Casp2^−/−^* and *Casp2^C320S^* mice (*n* = 5 independent liver samples per genotype). Stain-free membrane (SFM) indicates protein loading. Protein expression levels quantitated from (E) are shown as fold change from expression levels in *Casp2^+/+^* liver lysates, relative to total protein loading. Values are means ± SEM. **P < 0.01.

Quantitation of levels of key liver enzyme biomarkers in plasma from 12-month-old mice found elevated levels of alanine aminotransferase (ALT), aspartate aminotransferase (AST), and lactate dehydrogenase (LDH), particularly in *Casp2^C320S^* mice at this time, compared with WT mice (Fig. 3A). Further, increases were observed in the >18-month-old tumor-free cohort with a higher trend for liver damage markers in *Casp2^C320S^* mice compared to *Casp2^+/+^* mice (Fig. 3A). In addition to these plasma biomarkers, livers from *Casp2^C320S^* mice had higher levels of oxidative damage by 12 months of age, as detected by increases in lipid peroxidation (malondialdehyde, MDA assay) and protein carbonylation (Fig. 3B). This oxidative liver damage is associated with increased oxidative DNA damage, detected by 8-hydroxyguanosine (8-OHdG) staining (Fig. 3C). Increased lipid peroxidation is often a consequence of increased ferroptosis, and consistent with this, we observed an increase in hepatic iron content (detected by Perls Prussian blue staining) (Fig. 3D), together with reduced levels of glutathione peroxidase 4 (GPX4), a key antioxidant and ferroptosis inhibitor (Fig. 3E). These features suggest that loss of caspase-2 enzymatic activity promotes ferroptosis-mediated liver damage via GPX4 reduction or inactivation.

To further assess the possible role of caspase-2 in ferroptosis in liver cells, we used small interfering RNA (siRNA) to knockdown *CASP2* in Huh7 hepatoma cells and then induced ferroptosis with either erastin or RSL3, a small-molecule inhibitor of GPX4. Consistent with recent findings in lung cancer cell lines (*42*), *CASP2* depletion further sensitized Huh7 cells to ferroptosis, which is partly rescued by addition of ferrostatin (Fer1), an inhibitor of lipid peroxidation (fig. S4A). As expected, ferroptosis induction led reduced levels of GPX4 in control siRNA interference (siRNAi)–treated Huh7 cells and reduced cell viability (fig. S4, A and B). However, *CASP2* knockdown markedly reduced levels of GPX4 compared to control siRNAi-treated cells, even in the absence of ferroptosis-inducing drugs (fig. S4A). As a consequence, *CASP2*-depleted cells show increased sensitivity to ferroptosis-mediated cell death (fig. S4B). These data are consistent with a previous report (*42*) and further demonstrate that loss of caspase-2 can increase the sensitivity to ferroptosis by reducing GPX4 protein levels.

### Aging *Casp2^C320S^* mice develop steatosis

**Fig. 4.**
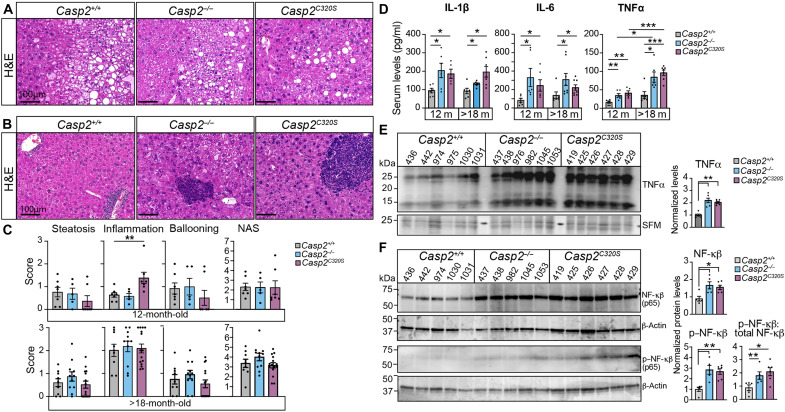
Steatosis and inflammation in *Casp2^C320S^* livers. (**A** and **B**) Representative images of H&E-stained liver sections from 12-month-old *Casp2^+/+^*, *Casp2^−/−^*, and *Casp2^C320S^* mice, showing areas of steatosis (A) and immune cell infiltration (B). Scale bars, 100 μm. (**C**) NAS scoring from 12- and >18-month-old *Casp2^+/+^*, *Casp2^−/−^*, and *Casp2^C320S^* livers as the sum of numerical scores for steatosis, hepatocellular ballooning, and lobular inflammation. Values are means ± SEM; *n* = 5 to 7 liver sections (mice per genotype). Significance is indicated as **P < 0.01. (**D**) Increased plasma IL-1β, IL-6, and TNFα in caspase-2-deficient mice. Values are means ± SEM; *n* = 5 to 6 mice per genotype. Significance is indicated as *P < 0.05 and ***P < 0.001. (**E** and **F**) Immunoblot of TNFα protein levels (E), total NF-κβ (p65) and phospho–NF-κβ (p65) protein levels (F) in liver lysates from 12-month-old mice. *n* = 5 to 6 independent liver samples per genotype are shown. SFM and β-actin are shown to indicate protein loading. Protein expression levels quantitated from (E) and (F) are shown as fold change from expression levels in *Casp2^+/+^* liver lysates, relative to protein loading. Values are means ± SEM. Significance is indicated as *P < 0.05 and **P < 0.01.

Loss of caspase-2 has been shown to protect young mice from high-fat or high-fructose diet–induced obesity and hepatosteatosis (*27*–*29*, *32*). While polyploidization can be protective against the development of steatohepatitis (MASLD) (*3*), pathologic polyploidization is associated with progression of MASLD and often HCC (*4*, *5*, *37*). We assessed the incidence of age-related steatosis and inflammation in standard laboratory diet–fed caspase-2-deficient mice (Fig. 4, A and B). Both *Casp2^−/−^* and *Casp2^C320S^* mice exhibited steatosis with aging (Fig. 4A). We noted that the incidence of steatosis by 12 months of age is reduced in *Casp2^C320S^* mice [9%; *X*^2^ (1, *N* = 15) = 10.97, *P* < 0.001] and somewhat lower (albeit nonsignificant) in *Casp2^−/−^* (17.6%), when compared with steatosis incidence in age-matched *Casp2^+/+^* mice (27%) (fig. S5A). However, older nontumor-bearing *Casp2^−/−^* and *Casp2^C320S^* mice (>18 months old) showed similar frequency of age-related steatosis to *Casp2^+/+^* mice (fig. S5A).

To further examine differences in the severity of steatosis, we assessed the nonalcoholic fatty liver disease (NAFLD) activity score (NAS) from H&E-stained liver sections. No significant difference in NASs is observed between genotypes at 12 months or even at >18 months of age (Fig. 4C). Together, these findings demonstrate that while younger caspase-2-deficient mice show some protection from hepatic steatosis, loss of caspase-2 does not prevent age-related steatosis incidence or severity.

### Loss of caspase-2 activity augments chronic liver inflammation

At 12 months of age, the most discernible histological difference in livers from *Casp2^C320S^* mice is an increased incidence and severity of inflammation, confirmed with NAS scoring of lobular inflammation (Fig. 4, B and C). Macroscopic analyses of H&E-stained sections showed marked early periportal lymphocytic and lymphomatous infiltration as well as lobular immune cell infiltrates in livers from both *Casp2^C320S^* mice (64%;14 of 22 mice) and *Casp2^−/−^* mice (47%; 8 of 17 mice) at 12 months of age (Fig. 4B and fig. S5B). In comparison, 33% WT mice (5 of 15) showed only minor areas of portal and periportal lymphocytic infiltration at this age. These findings also indicate that enzymatic inactivation of caspase-2 further exacerbates age-associated hepatic inflammation compared to complete genetic ablation of *Casp2*.

Cytokine profiling of plasma from *Casp2^C320S^* mice showed increases in the levels of several proinflammatory chemokines and cytokines (fig. S5, C and D), including interleukin-1β (IL-1β), IL-6 and tumor necrosis factor–α (TNFα) by 12 months of age (Fig. 4, D and E). While *Casp2^C320S^* mice showed a more pronounced increase in hepatic inflammation, both *Casp2^−/−^* and *Casp2^C320S^* samples had comparable increases in serum proinflammatory cytokines at both age groups that may suggest general systemic inflammation following loss of caspase-2. Levels of TNFα were also further increased by 18 months of age in tumor-free mice, consistent with a chronic inflammatory phenotype observed in caspase-2-deficient mice (Fig. 4D). The higher TNFα levels were proportionate to increased levels of nuclear factor κβ (NF-κβ), as well as phosphorylated NF-κβ, in both *Casp2^−/−^* and *Casp2^C320S^* livers (Fig. 4F), implicating a role for NF-κβ in regulating inflammation in caspase-2-deficient mice. These findings also indicate that early inflammation precedes steatosis in *Casp2^C320S^* mice and suggestive of hepatitis-induced age-related liver damage and neoplastic phenotype.

### Increased activation of hepatic stellate cells drives liver fibrosis in *Casp2^C320S^* mice

**Fig. 5.**
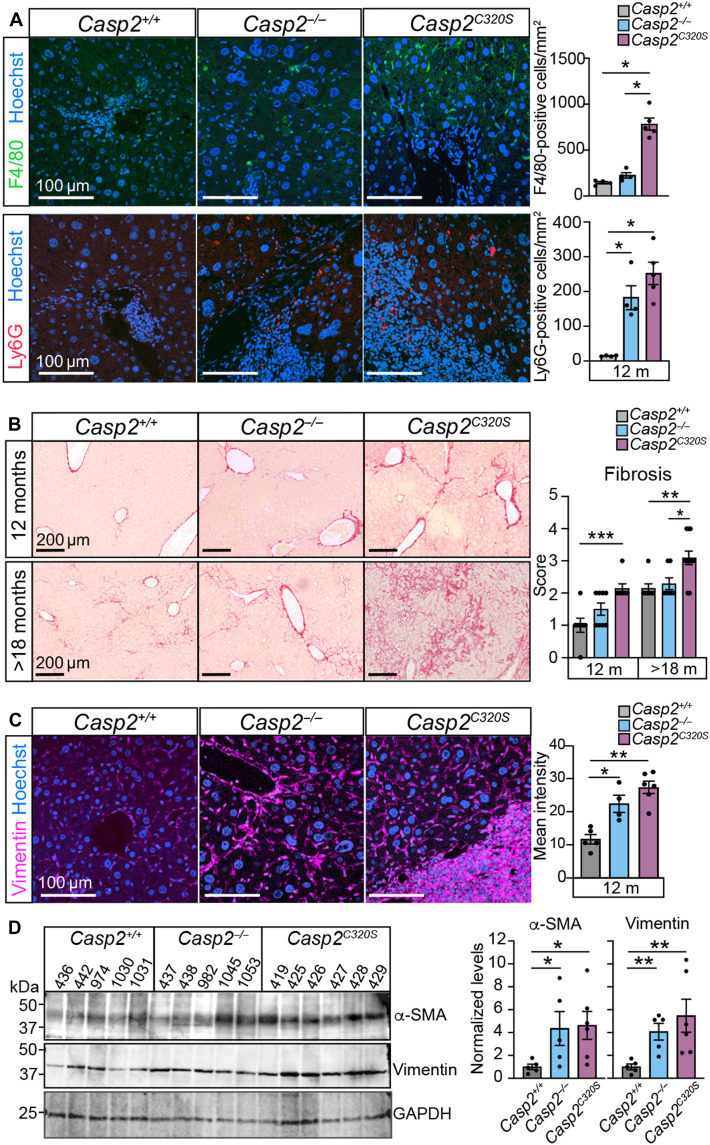
Livers from aging *Casp2*^*C320S*^ mice exhibit increased fibrosis. (**A**) Representative immunofluorescent images of liver sections stained with F4/80 macrophages (green) and Ly6G neutrophils (red) merged with Hoechst (blue). Quantitation of F4/80- and Ly6G-positive cells is shown. Values are means ± SEM; *n* = 4 to 5 independent liver samples. Significance is indicated as *P < 0.05. (**B**) Representative images of Picrosirius red stained liver sections from 12- and >18-month-old *Casp2^+/+^*, *Casp2^−/−^*, and *Casp2^C320S^* mice. Scale bars, 100 μm. Quantitation of liver fibrosis score in mice is shown. Values are means ± SEM; *n* = 6 to 7 independent mouse livers. Significance is indicated as *P < 0.05, **P < 0.01, and ***P < 0.001. (**C**) Representative immunofluorescent images of liver sections stained with vimentin (magenta) and Hoechst (blue). Scale bars, 100 μm. Quantitation represents mean fluorescence intensity (in pixels) per FOV across five FOVs per sample. Values are means ± SEM, and significance indicated as *P < 0.05 and **P < 0.01. (**D**) Immunoblot showing increased vimentin and α–smooth muscle actin (α-SMA) in liver lysates from *Casp2^+/+^*, *Casp2^−/−^*, and *Casp2^C320S^* mice (*n* = 5 independent liver samples per genotype). Glyceraldehyde-3-phosphate dehydrogenase (GAPDH) indicates protein loading control. Protein expression levels quantitated from (D) are shown as fold change from expression levels in *Casp2^+/+^* liver lysates, relative to GAPDH levels. Values are means ± SEM. Significance is indicated as *P < 0.05 and **P < 0.01.

Inflammation, observed as immune cell infiltration, of lymphocytes, macrophages, and neutrophils plays a key role in promoting liver fibrosis (*43*). While neutrophils can induce damage via production of reactive oxygen species, resident hepatic macrophages (Kupffer cells) can also activate hepatic stellate cells (HSCs) and promote early fibrosis through proinflammatory immune cell recruitment (*43*). Immunostaining for these key immune cell infiltrates demonstrated accumulation of macrophages (F4/80^+^ cells) in *Casp2^C320S^* livers and increased number of infiltrating Ly6G^+^ neutrophils in livers from both *Casp2^−/−^* and *Casp2^C320S^* mice at 12 months of age (Fig. 5A). These findings demonstrate that the composition of the periportal and lobular immune cell infiltration in caspase-2-deficient livers includes macrophages and neutrophils.

Inflammation-induced liver disease progression driven by hyperpolyploidy and/or by MASLD promotes development of liver fibrosis and cirrhosis that can increase susceptibility to liver cancer (*4*). To assess liver fibrosis, we stained liver sections with Picrosirius red to detect collagen deposition in livers from 12- and >18-month-old tumor-free mice (Fig. 5B). Consistent with the increased liver damage at 12 months of age, the *Casp2^C320S^* livers showed increased levels of perisinusoidal fibrosis and periportal fibrosis compared with age-matched WT and *Casp2^−/−^* livers (Fig. 5B). The frequency and extent of fibrosis is further increased with aging, with extensive bridging fibrosis observed in several *Casp2^C320S^* livers at >18 months of age (Fig. 5B). Histological fibrosis scoring further demonstrated increased severity of fibrosis in *Casp2^C320S^* livers from 12 months of age compared to livers from both WT and *Casp2^−/−^* mice (Fig. 5B).

Liver damage and fibrosis are positively associated with the activation of HSCs (*44*). To examine the contribution of activated HSCs to the extent of liver inflammation and fibrosis, we immunostained liver sections for vimentin and observed increased levels in both *Casp2^−/−^* and *Casp2^C320S^* livers at 12 months of age, particularly around the periportal areas and areas marked by immune cell infiltration (Fig. 5C). Immunoblotting further demonstrated robust increases in both vimentin and α–smooth muscle actin (α-SMA) levels in caspase-2-deficient liver lysates (Fig. 5D), indicative of increased HSC activation that can contribute to hepatic fibrogenesis at this age. Together with HSC activation, the infiltration of lymphocytes, macrophages, and neutrophils all plays cooperating roles in promoting early fibrosis in *Casp2^C320S^* livers.

### Hepatocyte proliferation and senescence are increased in *Casp2^C320S^* livers

**Fig. 6.**
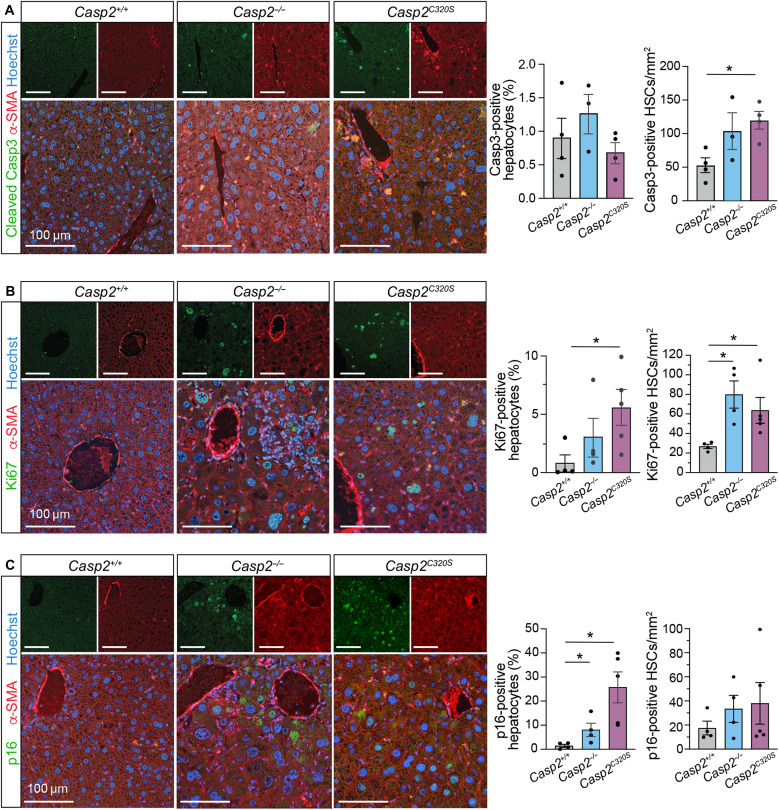
Increased proliferation and senescence in caspase-2-deficient livers. Representative immunostaining images of liver sections from 12-month-old *Casp2^+/+^*, *Casp2^−/−^*, and *Casp2^C320S^* mice, with (**A**) cleaved caspase-3 (Casp3, green), (**B**) Ki67 (green), and (**C**) p16 (green) costained with α-SMA (red) and Hoechst DNA stain (blue). Quantitation of positively stained hepatocytes (%) and HSCs (number cells per square millimeter) is indicated for each marker. Values are means ± SEM; *n* = 4 to 5 liver samples. Significance is indicated as *P < 0.05.

To examine the fate of polyploid hepatocytes in caspase-2-deficient livers, we assessed cell death, proliferation, and senescence by immunofluorescence in liver sections from 12-month-old mice. There were minimal levels of hepatocyte apoptosis, as detected by cleaved caspase-3 immunofluorescence, in livers from all mice of each genotype at this age (Fig. 6A). This indicates that although there is an increase in polyploid hepatocytes in caspase-2-deficient livers, these abnormally enlarged cells are not undergoing apoptosis. Where cleaved caspase-3 immunofluorescence is observed, this is also mostly associated with HSCs (Fig. 6A), as detected by costaining with α-SMA. This feature is suggestive of the activation-induced cell death of HSCs that has been reported during liver injury (*44*).

Increased hepatocyte proliferation in caspase-2-deficient livers is detected by staining with Ki67 (Fig. 6B). Notably, several of the Ki67-positive proliferating cells had enlarged nuclei or multiple nuclei, indicating that polyploid hepatocytes are able to continue dividing and accumulate in aging livers from *Casp2^−/−^* and *Casp2^C320S^* mice. The Ki67-positive hepatocytes with smaller nuclei or cells that are binucleate may also represent a proportion of diploid hepatocytes with regenerative capacity in these damaged livers. In addition to hepatocytes, we observed an increase in the number of proliferating HSCs, costained with α-SMA, in the absence of caspase-2 (Fig. 6B). These findings suggest that the enhanced collagen deposition and fibrosis in liver from caspase-2-deficient mice may be due to the increased activation of HSCs.

While proliferating hepatocytes are associated with regeneration in damaged livers, increased senescence also has a causative role in MASLD and liver disease progression (*45*). The role and contribution of hepatocyte senescence to the observed liver damage in caspase-2-deficient mice are assessed by immunostaining with the senescent marker p16^INK4A^. Consistent with the extent of liver damage observed, there is an increase in the number of senescent hepatocytes, particularly in livers from *Casp2^C320S^* mice at 12 months of age compared to liver tissue from *Casp2^+/+^* mice (Fig. 6C). *Casp2^C320S^* livers exhibited markedly greater number of p16-positive senescent hepatocytes compared with *Casp2^C320S^*-proliferating hepatocytes (mean = 25.78% in Fig. 6C compared to 5.58% in Fig. 6B). However, there is no increase in senescent HSCs in caspase-2-deficient livers compared with *Casp2^+/+^* mouse livers (Fig. 6C). Our findings suggest insufficient clearance or removal of senescent hepatocytes in the absence of caspase-2 that can further contribute to the observed age-related liver damage phenotype. These data also affirm that caspase-2 activity is required for apoptotic removal of both senescent cells and hyperpolyploid/aneuploid hepatocytes during aging.

### Proteomic analysis identifies an early hyperpolyploidy protein signature in liver from *Casp2^C320S^* mice

To define the early and age-related liver proteome changes that contribute to early hyperpolyploidy and enhanced liver damage in *Casp2^C320S^* mice, we analyzed and compared the global liver proteome from *Casp2^+/+^*, *Casp2^−/−^*, and *Casp2^C320S^* mice at 3, 12, and >18 months of age (before tumor observation). Proteomic analyses quantified 7140 hepatic proteins across all genotypes and age groups with principal components analysis, showing that the proteome is strongly influenced by both age and genotype (fig. S6, A to C). There are many common and distinct changes in protein abundance when comparing [*Casp2^C320S^* versus *Casp2^+/+^*] with [*Casp2^−/−^* versus *Casp2^+/+^*] liver proteomes at each age cohort (fig. S6D and tables S1 and S2). Notably, the differentially abundant liver proteins detected at each age are mostly nuclear proteins, with altered abundance of mitochondrial and endoplasmic reticulum (ER) proteins detected in older livers (12 months and >18 months), commonly associated with liver disease progression (fig. S6E).

**Fig. 7.**
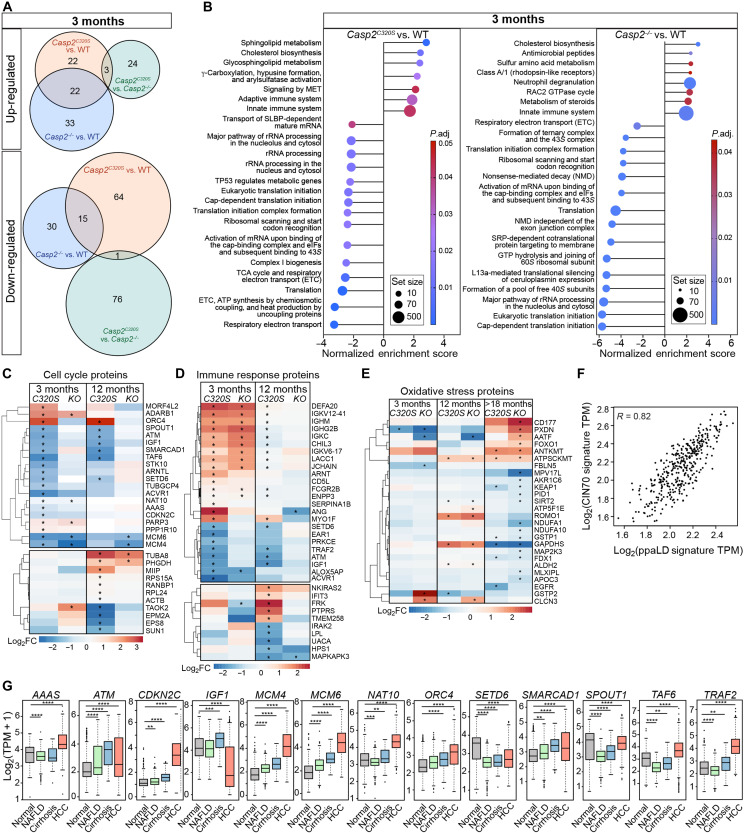
A pathogenic polyploidy protein signature identified in the caspase-2-deficient liver proteome. (**A**) Venn diagram summary of distinct and overlapping proteins that are significantly up-regulated or down-regulated in the indicated comparison groups, from 3-month-old liver samples. (**B**) GSEA of the top 15 enriched Reactome pathways associated with up-regulated and down-regulated genes in *Casp2^C320S^* versus WT (*Casp2^+/+^*) and in *Casp2^−/−^* versus WT (*Casp2^+/+^*) livers at 3 months. rRNA, ribosomal RNA. (**C** to **E**) Heatmaps showing increased (red) and decreased (blue) expression [log_2_ fold change (log_2_FC)] of proteins associated with (C) cell cycle, (D) immune system, and (E) oxidative stress (from the mouse MSigDB) and sorted by significantly altered proteins in *Casp2^C320S^* versus *Casp2^+/+^* livers at 3 months (top) or 12 months (bottom). Significantly altered proteins (adjusted *P* < 0.05) are indicated with an asterisk (*). Oxidative stress proteins were sorted by significantly altered proteins in *Casp2^−/−^* versus *Casp2^+/+^* livers at the indicated age groups. KO, knockout. (**F**) Correlation analysis between the CIN70 gene signature and ppaLD gene signature in liver cancer (LIHC-TCGA). Statistical comparisons were performed using Pearson correlation coefficient. TPM, transcripts per million. (**G**) Gene expression comparisons of the indicated human genes from the ppaLD gene signature, comparing expression in normal, NAFLD, cirrhosis, and HCC liver samples (GepLiver). Box and whisker plots represent the interquartile range and median value. Statistical comparisons were performed using nonparametric Wilcoxon rank-sum tests with significance indicated as **P < 0.01, ***P < 0.001, and ****P < 0.0001.

Analysis of proteomes from 3-month-old livers identified 47 significantly up-regulated and 80 down-regulated proteins in *Casp2^C320S^* livers compared to *Casp2^+/+^* samples (Fig. 7A and table S2A), with several distinct changes to the age-matched *Casp2^−/−^* liver proteomes (fig. S7, A and B, and table S2, A to C). Gene set enrichment analysis (GSEA) of differentially abundant proteins in 3-month-old *Casp2^C320S^* livers indicated that immune system, cholesterol biosynthesis, lipid metabolism, and MET-signaling were up-regulated, whereas pathways associated with p53 regulation of metabolic genes, mRNA processing and transport, translation, and electron transport chain (ETC) were down-regulated from a young age (Fig. 7B and table S3A). Several similarities in GSEA were detected in both *Casp2^C320S^* and *Casp2^−/−^* livers, with up-regulation of mitochondrial translation processes at 12 months, suggestive of misregulated translation that is frequently associated with impaired oxidative phosphorylation and mitochondrial fidelity that can drive liver disease progression (fig. S7, C and D, and table S3, A to F). Consistent with caspase-2 role in DNA damage and MC, older *Casp2^C320S^* livers showed enrichment of proteins involved in DNA damage repair, cell cycle, and ploidy control (i.e., activation of the prereplicative complex, activation of ATR in response to replication stress, mitotic-G_1_ phase, G_1_-S transition, and assembly of the prereplicative complex) (fig. S7, C and D, and table S3, C to F). Notably, the altered proteins in *Casp2^−/−^* livers at >18 months were mostly associated with reduced p53 stabilization, p53-dependent DNA damage checkpoints, and G_1_-S transition, consistent with caspase-2 role in regulating these processes following age-related cellular stress (fig. S7D and table S3F).

Heatmap visualization highlighted the distinct protein signatures in young *Casp2^C320S^* livers, associated with increased hyperpolyploidy and susceptibility to liver damage including dysregulation of several cell cycle proteins (e.g., reduced AAAS, ACVR1, ATM, BMAL1, CDKN2C, MCM4, MCM6, NAT10, STK10, SPOUT1, TAF6, TUBGCP4, increased ADARB1, PPP1R10, and MORF4L2) at 3 and 12 months of age (Fig. 7C). In addition, we observe dysregulation of over 20 proteins involved in immune response pathways in both age cohorts [e.g., increased immunoglobulin (Ig) heavy chains, Igκ chains, IgJ chain, IGHM, IGHG2B, IGKC, IGKV12-41, CHIL3, ENPP3, FCGR2B, CD5L, ARNT, and SERPINA1B] (Fig. 7D). Notably, while only a few early changes in oxidative stress proteins were observed at 3 and 12 months, altered levels were prevalent in older (>18-month) *Casp2^−/−^* livers [e.g., KEAP1, GSTP1, GAPDHS, MLXIPL, FDX1, SIRT2, NDUFA1, NDUFA10, AKR1C6, MPV17L, FOXO1, and PXDN], consistent with a dysregulated oxidative stress response in aging caspase-2-deficient mice (Fig. 7E).

The dysregulated cell cycle proteins were mostly less abundant/down-regulated in *Casp2^C320S^* livers, with many proteins similarly dysregulated at both 3 and 12 months of age and strongly associated with instructing hyperpolyploidy in caspase-2-deficient livers (Fig. 7, C and D). On the basis of their role(s) in cell cycle control or DNA damage repair, altered expression in *Casp2^C320S^* livers at both 3 and 12 months, reported functions in chromosome instability, and/or strong positive correlation with the chromosome instability 70-gene (CIN70) signature (*46*) (Fig. 7F), we defined a distinct set of 13 proteins associated with early hyperpolyploidy caused by loss of caspase-2 activity [i.e., AAAS, ATM, CDKN2C, IGF1, MCM4, MCM6, NAT10, ORC4, SETD6, SMARCAD1, SPOUT1/C9orf114, TAF6, and TRAF2). We termed this protein signature the “ppaLD” signature. Some of these proteins have roles in both cell cycle and immune regulation, reflecting a critical interplay between hyperploidy and immune homeostasis. We assessed the associations of the gene expression for this ppaLD signature in liver disease and liver cancer and found that alterations in each of these genes are observed in both HCC and hepatobiliary cancer (HBC) (albeit at low frequency) (fig. S8A). Gene expression for each of these 13 candidates is significantly dysregulated in liver disease (MASLD and cirrhosis) and/or HCC (Fig. 7G), with dysregulated levels of several of these genes associated with overall patient survival in HCC (i.e., *CDKN2C*, *IGF1*, *MCM4*, *MCM6*, *NAT10*, *ORC4*, *SMARCAD1*, *TAF6*, and *TRAF2*) (fig. S8B). These data provide a distinct set of putative clinical biomarkers associated with early hyperpolyploidy and aneuploidy that maypredict susceptibility to early liver disease onset, HCC, prognosis, or outcome.

**Fig. 8.**
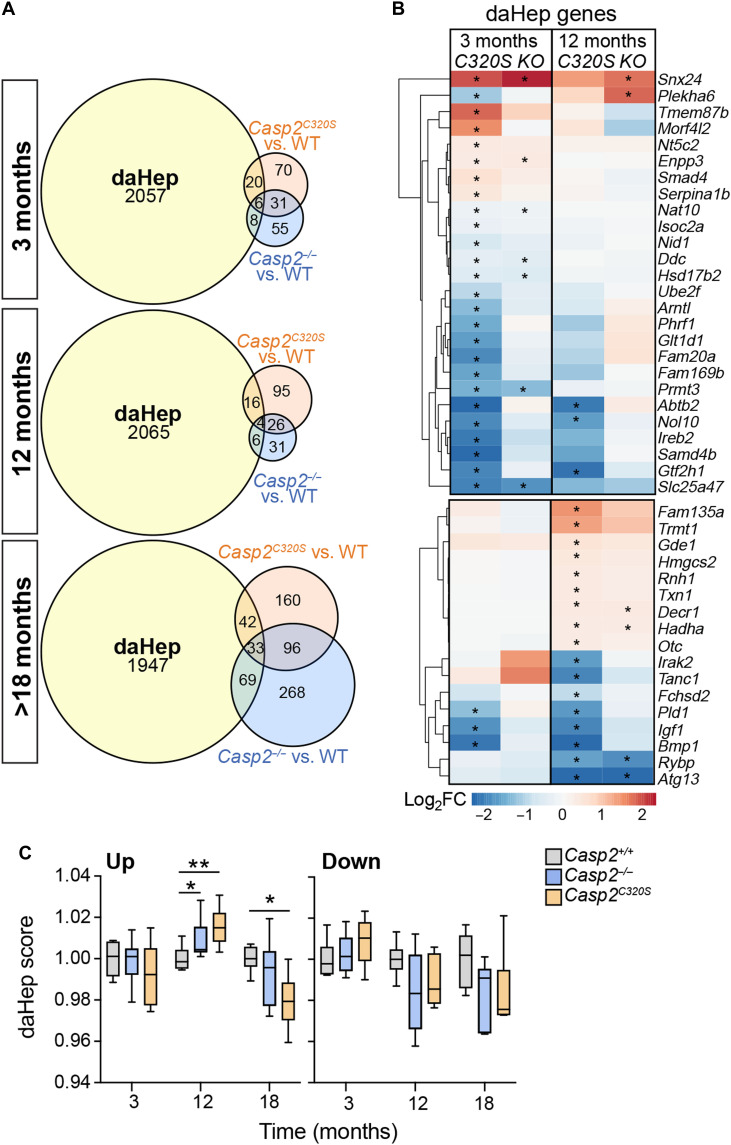
Comparisons between the caspase-2-deficient liver proteomes and the daHep signature. (**A**) Venn diagram comparing significantly altered proteins in *Casp2^C320S^* versus WT (*Casp2^+/+^*) and *Casp2^−/−^* versus WT (*Casp2^+/+^*) liver samples, with the daHep signature for each age cohort. (**B**) Heatmap illustrating altered expression (Log_2_FC) of proteins in *Casp2^C320S^* versus WT (*Casp2^+/+^*) livers that are common to the daHep gene signature, at 3 months (top) or at 12 months (bottom). Asterisk (*) indicates all significantly altered proteins with adjusted *P* < 0.05. (**C**) daHep gene signature score analysis. Normalized protein expression from *Casp2^C320S^*, *Casp2^−/−^*, and *Casp2^+/+^* (WT) liver samples (*n* = 6 per genotype) at the indicated age groups are used to compute daHep gene signature enrichment score for each sample using manually curated up- and down-regulated daHep-associated gene sets via single-sample GSEA (ssGSEA), respectively. Statistical comparisons between groups are performed using nonparametric Wilcoxon rank-sum tests. *P < 0.05; **P < 0.01.

To assess whether the early and age-related proteomic changes in *Casp2^C320S^* livers are similar or distinct to gene expression alterations associated with MASLD progression, we also compared the liver proteome changes from caspase-2-deficient mice to the disease-associated hepatocyte (daHep) gene signature (*47*). Livers from 3-month-old *Casp2^C320S^* mice show dysregulation of 26 proteins that constitute the daHep signature and that are distinct from the ppaLD signature and highlight MASLD susceptibility genes at an early stage (Fig. 8, A and B, and table S4A). Similarities to the daHep signature were also observed at 12 months of age, a time when we observe extensive inflammation and fibrosis in *Casp2^C320S^* livers but more prominently in older livers from caspase-2-deficient mice, consistent with increased metabolic dysfunction with aging (Fig. 8, A and B). We next scored the daHep enrichment from both up-regulated and down-regulated genes independently and plotted the enrichment score as fold change compared to *Casp2^+/+^* samples (Fig. 8C and table S4B). Our data show that the up-regulated proteins in 12-month-old livers from *Casp2^C320S^* and *Casp2^−/−^* mice are more positively associated with the daHep signature compared to age-matched *Casp2^+/+^* mice and include proteins associated with dysregulated lipid metabolism and fatty acid oxidation that are predictive of HCC susceptibility (HADHA, DECR1, GDE1, HMGCS2, OTC, RNH1, TRMT1, FAM135A, and TXN1) (Fig. 8C and table S4A).

These data indicate that, while aging caspase-2-deficient mice show some similarities in liver phenotypes and gene/protein expression associated with MASLD, pathogenic hyperpolyploidy–mediated liver disease is distinctly caused by the dysregulated levels of ppaLD proteins and is primarily associated with inflammatory-mediated liver disease. Notably, there is also no significant positive correlation with the daHep disease signature at 3 months or even at >18 months, further indicating that the proteins driving early hyperpolyploidy in caspase-2-deficient mice are mostly distinct from those that drive MASLD-mediated HCC.

## DISCUSSION

A role for caspase-2 in controlling aneuploidy and polyploidy has been demonstrated in various cell lines, primary cell types, and mouse tissues and is a proposed mechanism for caspase-2-mediated tumor suppression (*16*, *21*, *22*). In hepatocytes, increased polyploidy caused by caspase-2 loss is associated with organogenesis liver homeostasis but appears to have opposing roles in protecting against carcinogen-induced HCC (*3*, *24*). This study now establishes the essential role of caspase-2 proteolytic activity in regulating hepatocyte polyploidy and important role in constraining deleterious ploidy alterations, marked by hyperpolyploidy and aneuploidy, in both young and aging mice. Our findings underscore the significance of caspase-2 enzymatic function in maintaining age-related liver homeostasis and protecting against hepatitis-induced liver pathology and HCC.

We previously demonstrated a role for caspase-2 in limiting aneuploidy in the bone marrow compartment during aging in mice and in primary mouse splenocytes exposed to antimitotic agents, highlighting its important role in limiting GIN in different cell types (*14*, *18*). Furthermore, we established that caspase-2 is required for apoptotic deletion of cells with mitotic defects to limit aneuploidy (*14*, *48*, *49*). Consistent with this, our data now show that loss of caspase-2 activity promotes the survival and accumulation of hepatocytes with hyperpolyploidy (predominantly mononucleated polyploidy) and aneuploidy. As a consequence, persistent and unresolved hyperpolyploidization with aging disrupts hepatocyte polyploidy dynamics (*4*) and promotes GIN, age-related oxidative liver damage, and spontaneous HCC development.

In addition to limiting polyploidy via MC signaling, caspase-2 role in liver homeostasis is mediated by additional functions in regulating metabolism and fat homeostasis (*7*, *13*). Hence, loss of caspase-2 is liver protective in young mice by promoting lipogenesis and preventing high-fat/fructose diet–induced steatohepatitis (*26*, *29*). Our data suggest that caspase-2 catalytic activity is partly required for its function in fat metabolism, with *Casp2^C320S^* mice having reduced fat accumulation in adipose tissue (both gWAT and dWAT) and reduced age-related liver steatosis incidence at 12 months. However, our findings importantly demonstrate that loss of caspase-2 does not completely prevent hepatosteatosis with aging, despite it offering protection in younger mice. Our data also suggest that chronic liver inflammation and disease in 12-month-old caspase-2-deficient mice are partly independent of MASLD. Notably, our proteomic data also show that aging caspase-2-deficient livers also have a higher daHep score at 12 months compared to their *Casp2^+/+^* mice, highlighting several common genes/proteins that drive age-related liver damage and disease in these distinct models. This includes the up-regulation of several proteins in young caspase-2-deficient mice (HADHA, DECR1, GDE1, HMGCS2, OTC, RNH1, TRMT1, FAM135A, and TXN1), associated with dysregulated lipid metabolism and fatty acid oxidation, which may serve as early predictive markers of susceptibility to MASH and HCC. Notably, our findings also highlight different age-related mechanisms driving HCC in the absence of caspase-2 and clear distinctions in the different models used to analyze hepatosteatosis, with MASH models driven by a high-fat or high-fructose diet that trigger a chronic ER stress–related response, dysregulated hepatic lipid metabolism, fat accumulation, inflammation, and fibrosis (*29*, *47*). In contrast, aging is associated with multiple dysregulated pathways including reduced cellular and DNA damage repair, cellular senescence, GIN, and metabolic and mitochondrial dysfunction (*50*). Critical to this understanding, we have demonstrated that hepatocyte hyperploidy in young caspase-2-deficient mice is marked by dysregulation of a distinct set of 13 proteins (AAAS, ATM, CDKN2C, IGF1, MCM4, MCM6, NAT10, ORC4, SETD6, SMARCAD1, SPOUT1/C9orf114, TAF6, and TRAF2) associated with early onset pathogenic hyperpolyploidization that correlate with increased CIN, liver disease progression, and patient survival in HCC (*3*, *4*).

In addition to early hyperpolyploidy, the increase in infiltrating immune cells and severity of inflammation by 12 months of age in *Casp2^C320S^* livers is a significant feature of early liver damage and hepatitis-driven liver injury in caspase-2-deficient mice. The liver proteome demonstrates that young mice (3 months old) already exhibit immune dysregulation, including increased immunoglobulin isotypes and variants, B cell markers, and macrophages. These data suggest that hyperpolyploidy may trigger dysfunctional immune responses at an early age, which increase the incidence of chronic inflammation. An apoptosis-independent role for caspase-2 in inflammation and immune regulation has also previously been suggested (*51*). Thus, we cannot rule out the possibility that caspase-2 exerts distinct regulatory effects on both hepatocyte ploidy and inflammatory pathways, which may synergistically contribute to the initiation and progression of liver disease.

Liver inflammation is evident in both *Casp2^−/−^* and *Casp2^C320S^* aged mice but is elevated in *Casp2^C320S^* mice at 12 months of age, marked by increased macrophage activation (e.g., Kupffer cells), together with activation of HSCs and neutrophils. While macrophages are key inflammatory drivers of fibrogenesis, neutrophils contribute to elevated reactive oxygen species and IL-1β–driven inflammation (*43*). Activation of HSCs occurs in response to both chronic inflammation and/or oxidative stress and responsible for deposition of extracellular matrix in the liver (*44*). Thus, the elevated inflammatory phenotype in caspase-2-deficient mice is a key driver of liver fibrosis. Furthermore, the marked increase in hepatocyte senescence in caspase-2-deficient livers is a key cellular mechanism linking chronic liver injury to fibrotic progression during aging. Senescent hepatocytes also release inflammatory factors that further activate macrophages and HSCs, thus perpetuating a pathogenic cycle of hyperpolyploidy and inflammation that is elevated in *Casp2^C320S^* mice. Collectively, the accumulation of macrophages, impaired resolution of immune responses, and inefficient clearance of senescent hepatocytes represent converging mechanisms that drive chronic liver pathology and HCC development in aging *Casp2^C320S^* mice.

The increased oxidative liver damage is also more predominant in *Casp2^C320S^* mice and likely driven by infiltrating inflammatory cells, together with a dysregulated oxidative stress response caused by caspase-2 deficiency (*9*, *25*). As a consequence, increased oxidative stress is a key driver of ferroptosis in aging mice that can further exacerbate inflammation, MASLD, and tumorigenesis (*52*, *53*). Our findings are also partly consistent with a recently reported role for caspase-2 in negatively regulating ferroptosis (*42*). However, this study observed increased ferroptosis in caspase-2-deficient cells only when p53 is absent or mutated (p53^R273H^), that was independent of caspase-2 catalytic activity (*42*). In contrast, our data in the aging liver demonstrate that ferroptosis is augmented in *Casp2^C320S^* mice, highlighting a physiological role for caspase-2 enzymatic activity in restricting age-related ferroptosis in the liver. While increased ferroptosis may be caused by the chronic liver damage in caspase-2-deficient mice, further analysis is required to determine whether caspase-2 has a direct role in regulating ferroptosis and how or whether it directly effects GPX4 levels.

The heightened liver damage and histological differences in age-related progressive liver damage phenotypes between *Casp2^−/−^* and *Casp2^C320S^* mice suggest that the expression of the catalytic mutant protein may affect normal stress-induced caspase-2 signaling, possibly through a scaffolding function that can either obstruct or facilitate adaptor protein function (e.g., PIDD1 and RAIDD) or substrates (e.g., BID and MDM2). While expression of caspase-2-C320S mutant protein can impede MC-mediated MDM2-p53 signaling to increase hepatocyte polyploidy, our data also suggest that the absence of a robust MC response in the presence of persistent mitotic or DNA damage intensifies p53-mediated DNA damage response, increased p21- and p16-induced cell senescence, and accumulation of oxidative damage that can further fuel hepatocyte senescence and other modes of cell death (i.e., ferroptosis). While PIDDosome is a caspase-2-activating complex following MC-induced cytokinesis failure, there are also several PIDD1-independent caspase-2-interacting platforms that can be impeded by expression of caspase-2-C320S mutant, including TNF receptor complex components, TRAIL receptor complex, CD95 complex, and RFXANK (*7*, *15*, *54*). Sequestering of any of these components by the caspase-2 catalytic mutant would contribute to dysregulated inflammation and cell death responses and/or favor parallel signaling pathways, including NF-κB activation in immune cells. Alternatively, the enhanced liver damage phenotypes observed in *Casp2^C320S^* mice may highlight apoptosis-independent functions of caspase-2, including a recently reported role for caspase-2 as a deubiquitinase to maintain proteostasis and cellular ubiquitin homeostasis (*55*). While these signaling components were not significantly altered in abundance in the caspase-2-deficient liver proteomes, this does not rule out changes in protein modifications, activation status, and/or cell type–specific responses that may be influenced by mutant caspase-2 protein to potentiate inflammation and tumorigenesis with aging. Further examination of the caspase-2-C320S interactome in hepatocytes may help elucidate the underlying mechanisms.

Overall, this study establishes the critical role of caspase-2 enzymatic activity in regulating hepatocyte polyploidy and aneuploidy during liver aging. Notably, we demonstrate that early onset of hyperpolyploidy, karyomegaly, and aneuploidy in caspase-2-deficient young mice correlates with dysregulation of a distinct set of cell cycle proteins linked to heightened liver pathology and spontaneous age-related tumor formation. While short-term caspase-2 loss may promote polyploidy as a protective response to cellular stress, our findings caution against its long-term inhibition as a therapeutic strategy, given its contribution to pathogenic ploidy and liver tumorigenesis.

### Limitations

A key limitation of this study is the reliance on global *Casp2^*−/−*^* and mutant mouse models, which may obscure the hepatocyte-specific functions of caspase-2. Future investigations should aim to clarify the cell-autonomous role of caspase-2 and determine whether its activity in hepatocytes or potentially in other liver-resident cell types contributes to the observed phenotypes, including liver disease progression in caspase-2-deficient mice. In addition, our study does not resolve the complex roles of caspase-2 in mediating hyperpolyploidy-driven inflammation, oxidative stress, or ferroptosis, nor does it distinguish whether these processes are directly induced by polyploidy or regulated independently through other caspase-2 functions, especially given their tight interconnection (*3*, *4*, *7*, *13*). Further mechanistic studies are warranted to explore potential scaffolding functions of the caspase-2-C320S protein and to define its specific regulatory role in suppressing hyperpolyploidy and/or modulating alternative pathways.

## MATERIALS AND METHODS

### Animals, tissue collection, and histology

Female and male *Casp2^−/−^*, *Casp2^C320S/C320S^* (designated *Casp2^C320S^*), and WT (*Casp2^+/+^*) mice, both on a C57BL/6J background, have been described previously (*14*, *19*). The *Casp2^−/−^* and *Casp2^C320S^* mice were backcrossed to C57BL/6J inbred mice for ~10 to 15 generations and subsequently rederived into the same C57BL/6J mouse strain at the UniSA Core Animal Facility, ensuring genetic uniformity. WT (*Casp2^+/+^*) mice derived from the same breeding colonies (i.e., *Casp2^+/−^* or *Casp2^+/C320S^* heterozygous crosses) were then bred homozygously and progeny generated at approximately the same time and generation as *Casp2^−/−^* or *Casp2^C320S^* cohorts. Mice were grouped into three age cohorts: 3 to 4, 12, and 18 to 30 months. All experimental mice were cohoused and under pathogen-free conditions in individually ventilated cages, with a 12-hour light:dark cycle and fed ad libitum on standard chow. All procedures for animal experiments were approved and performed according to Animal Ethics Committee of University of South Australia (ethics numbers U05-16, U26-19, and U29-22) in accordance with the Australian code for the care and use of animals for scientific purposes [National Health and Medical Research Council (NHMRC) 8th Edition 2013, updated 2021]. Genotyping of mouse ear biopsies carried out by Transnetyx (www.transnetyx.com).

Mouse plasma was collected from anesthetized mice by cardiac puncture and processed in MiniCollect EDTA Tubes (Greiner) by centrifugation (2000*g* for 10 min), within 1 hour of collection. Plasma was aliquoted and stored at −80°C until analysis. Harvested tissues were fixed in HistoChoice (Sigma-Aldrich), and tissue processing, paraffin embedding, and sectioning were carried out by Adelaide University Histology Services. Freshly dissected tissues were snap-frozen in liquid nitrogen and stored at −80°C until analysis.

### Histological tissue examination

Tissue masses were independently assessed as neoplasms by UniSA veterinarians on macroscopic examination and histological characterization of tissue masses/tumor performed in the liver. Liver tissue sections (5 μm) were stained with H&E, with Picrosirius red (1 mg/ml; Direct Red 80 in picric acid, Sigma-Aldrich) or Perl’s Prussian blue (20% hydrochloric acid and 10% potassium ferrocyanide solution) counterstained with nuclear fast red (Sigma-Aldrich), based on standard established protocols. Slides were washed in distilled water (or with acidified water–0.5% acetic acid for Picrosirius red staining), dehydrated in a graded ethanol series (75, 80, 90, and 100%), cleared in xylene (2 × 3 min), and mounted in Depex mounting medium (Sigma-Aldrich). Stained slides were scanned at ×20 magnification on a NanoZoomer 2.0-HT Digital Slide Scanner (Hamamatsu Photonics), and images were analyzed using NDP.view2 (Hamamatsu).

#### NAFLD activity scoring

NAS is carried out using ×20 magnification images of liver sections stained with H&E-stained liver sections. Approximately 9 to 10 images per liver (per mouse) were scored from seven to eight mice per genotype per age cohort. NAS is based on levels of steatosis (lipidosis or fatty change; score, 0 to 3), inflammation (lobular inflammatory cell infiltration; score, 0 to 3), and hepatocyte ballooning (hepatocyte swelling/enlargement/ballooning degeneration; score, 0 to 2). Total NAS represents the sum of scores for steatosis, lobular inflammation, and ballooning (ranging from 0 to 8).

#### Fibrosis scoring

Liver fibrosis is assessed by intensity and pattern of Picrosirius red–stained liver sections and scored over 10 fields of view (FOVs) per tissue. The score is divided into 0 to 4 stages based on staining pattern: 0 = none; 1 = perisinusoidal/pericellular fibrosis around central veins (zone 3), focally or extensively present; 2 = zone 3 perisinusoidal/pericellular fibrosis with focal or extensive periportal fibrosis; 3 = zone 3 perisinusoidal/pericellular fibrosis and portal fibrosis with focal or bridging fibrosis; 4 = cirrhosis as defined by evidence of severe bridging fibrosis (fibrous septa) with abnormal hepatic nodules.

### Liver damage analysis

Lipid peroxidation was measured using the TBARS assay kit (Cayman Chemicals), and protein oxidation was measured using a protein carbonyl kit (Cayman Chemicals) according to the manufacturer’s instructions. Protein concentration from liver lysates was determined using a bicinchoninic acid (BCA) Protein Assay kit (Bio-Rad).

### Plasma biochemistry and enzyme-linked immunosorbent assay

Liver function tests (ALT, AST, and LDH activity) were determined in plasma by automated analysis (SA Pathology, Adelaide, Australia). Plasma IL-6, TNFα, and IL-1β levels were quantitated with commercially sourced mouse enzyme-linked immunosorbent assay kits (ABclonal).

### Immunohistochemistry and immunofluorescence

HistoChoice-fixed, paraffin-embedded liver sections were deparaffinized in xylene, rehydrated in a graded ethanol series (100, 95, 90, 80, 75, and 50%), and hydrated in distilled water. Antigen retrieval was performed by subboiling tissue sections in 0.1 M citrate buffer solution (pH 6.0) for 10 to 15 min and cooled on ice for 30 min. For immunohistochemical detection, endogenous peroxidase activity was blocked with 3% (v/v) H_2_O_2_/phosphate-buffered saline (PBS) solution for 15 min, and nonspecific antibody binding was blocked in 5% (v/v) fetal bovine serum/PBS solution with 0.1% Tween 20 (PBS-T) for 30 min. Tissue sections were then incubated with 8-OHdG (1:500 dilution) (ab48508, Abcam) overnight at 4°C, followed by incubation with biotinylated goat anti-mouse IgG secondary antibody (1:250) in blocking solution (GE Healthcare). Avidin/biotin complex reagent (VECTASTAIN Q8 ABC kit, Vector Labs) is added for 1 hour at room temperature, and peroxidase substrate (3,3′-diaminobenzidine) was added for color development (5 to 10 min). Tissue sections were dehydrated in a graded ethanol series and coverslips mounted in Depex mounting medium (Sigma-Aldrich). Cells with positive staining are scored in at least five FOVs and reported as means ± SEM (*n* = 4 to 5).

For immunofluorescence detection, following antigen retrieval, nonspecific antibody binding was blocked in 5% (v/v) goat serum in PBS-T for 30 min, and tissue sections were incubated with anti-GCP3 (1:200 dilution) (ab66596, Abcam), anti–cytokeratin 8 (1:200 dilution) (ab53280, Abcam), anti-Ki67 (1:500 dilution) (ab15580, Abcam), anti-F4/80 (1:100 dilution) (MF48000, Invitrogen), anti-Ly6G (1:200 dilution) (551459, BD Biosciences), anti-vimentin (1:100 dilution) (ab92547, Abcam), anti–α-SMA (1:200 dilution) (ab7817, Abcam), anti-p16 (sc1207, Santa-Cruz), or anti–cleaved Casp3 (#9669, Cell Signaling Technology) diluted in PBS-T overnight at 4°C. Sections were washed in PBS-T, followed by incubation with the appropriate Alexa Fluor–conjugated secondary antibody (1:500) in PBS-T for 1 to 2 hours at room temperature (GE Healthcare). Sections were counterstained with Hoechst 33342 (Thermo Fisher Scientific) and mounted in antifade (Thermo Fisher Scientific). Immunostained tissues were observed with a confocal laser scanning microscope LSM800 (Zeiss). Images were analyzed with ImageJ (National Institutes of Health) and positively stained cells quantitated from 8 to 10 FOVs (×20 magnification area = 319.45 mm by 319.45 mm) and expressed as % positive stained cells (from ~1000 cells) or number cells per square millimeter.

### Immunoblotting

Liver homogenates were made by mincing liver pieces (~50 to 100 mg of tissue) in radioimmunoprecipitation assay (RIPA) buffer [25 mM tris-HCl (pH 7.4), 150 mM NaCl, 1% NP-40, 1% sodium deoxycholate, and 0.1% SDS] supplemented with 1× Halt Protease and Phosphatase Inhibitor Cocktail, EDTA (Thermo Fisher Scientific). Homogenates were clarified by centrifugation at 16,000*g*, and protein concentration was determined with BCA (Bio-Rad). For immunoblot analysis, 50 μg of lysates were resolved on 4 to 20% Mini or Midi TGX stain-free precast SDS–polyacrylamide gel electrophoresis gels (Bio-Rad) in running buffer (250 mM tris, 192 mM glycine, and 0.06% SDS). Proteins were transferred onto polyvinylidene difluoride membrane (Bio-Rad) in transfer buffer (25 mM tris, 192 mM glycine, 20% methanol, and 0.05% SDS), and membranes were incubated in 5% (w/v) skim milk powder (Diploma) in TBS-T [20 mM tris, 150 mM NaCl (pH 7.4), 0.05% Tween 20] for 1 hour at room temperature, followed by incubation with the indicated primary antibodies overnight at 4°C: caspase-2 (clone 11B4), MDM2 (clone E3G5I) (#51541, Cell Signaling Technology), p53 (clone 1C12) (#2524, Cell Signaling Technology), phospho-p53 (Ser^15^) (#9284, Cell Signaling Technology), p21 (12D1) (#2947, Cell Signaling Technology), γH2AX (#25775, Cell Signaling Technology), cleaved caspase-3 (#9661, Cell Signaling Technology), NF-κβ p65/RelA (sc-372, Santa Cruz Biotechnology), phospho–NF-κβ p65/RelA (S468) (AP1460, ABclonal), TNFα (506314, BioLegend), β-actin (A1978, Sigma-Aldrich), vimentin (ab92547, Abcam), α-SMA (ab7817, Abcam), GPX4 (A21440, ABclonal), and GAPDH (#2118, Cell Signaling Technology). Following antibody incubation, membranes were washed in TBS-T and incubated with species-specific secondary antibodies conjugated to either horseradish peroxidase, alkaline phosphatase, or Cy5 for 1 hour at room temperature. Following membrane washes in TBS-T, blots were developed by enhanced chemiluminescence (ECL) with ECL Prime Western Blotting System reagents (Cytiva) and detection of luminescence signals on a Fuji LAS4000 System (GE Healthcare) or Chemidoc MP (Bio-Rad) or via enhanced chemifluorescence with AttoPhos reagent (Cytiva) and imaged on a Typhoon FLA 9000 (GE Healthcare). Cy5-conjugated antibodies were detected at excitation and emission wavelengths of 649 and 670 nm on a Typhoon FLA 9000 (GE Healthcare).

### Quantitative polymerase chain reaction analysis

Total RNA was isolated from liver tissue using TRIzol reagent (Life Technologies) and cDNA synthesized using oligo(dT) primers and high-capacity cDNA reverse transcription kit (Applied Biosciences). Real-time quantitative polymerase chain reaction (PCR) was performed using SYBR Green PCR Master Mix (QIAGEN) and analyzed as described previously (*16*), with all data normalized to *B-actin* levels. The following primer sets were used: *Mdm2*, GAAGGAGCACAGGAAAATATATGCA (forward) and GTCTGCTCTCACTCAGCGATGT (reverse);

*p21*, AGTGTGCCGTTGTCTCTTCG (forward) and ACACCAGAGTGCAAGACAGC (reverse); *Trp53*, CTCACTCCAGCTACCTGAAGA (forward) and AGAGGCAGTCAGTCAGTCTGAGTCA (reverse); β-*actin*, TGTTTGAGACCTTCAACACC (forward) and TAGGAGCCAGAGCAGTAATC (reverse).

### DNA content analysis

Hepatocytes were isolated from 3- to 4-month-old mice by established two-step collagenase perfusion of livers, followed by differential centrifugation, as previously described (*24*). Briefly, the liver was perfused with warm (37°C) Ca^2+^- and Mg^2+^-free Hank’s balanced salt solution (HBSS) from the inferior vena cava, followed by warmed liver digest medium (GIBCO) at a flow rate of 3 ml/min for 5 to 10 min. After perfusion, liver was isolated and gently agitated and pipetted in Dulbecco’s modified Eagle’s medium. The cell suspension was filtered through a cell strainer (100 μm^2^), hepatocytes were separated from nonparenchymal cells over three centrifugation steps at 50*g* for 5 min, and the cell pellet was resuspended in cold HBSS. For DNA content analysis of hepatocytes (*24*, *48*), harvested cells were washed with ice-cold PBS and fixed in 70% ice-cold ethanol in PBS, overnight at −20°C. Cells were centrifuged at 1200 rpm for 5 min and washed in PBS and then in PBS with 0.25% Triton X-100 (Sigma-Aldrich), followed by incubation in ~400 μl of staining solution [propidium iodide (25 μg/ml; Sigma-Aldrich) and ribonuclease A (40 μg/ml; Sigma-Aldrich)] for 2 hours at room temperature in the dark. Stained cells were stored at 4°C until flow cytometry analysis on a CytoFlex (Beckman Coulter). Approximately 10,000 cells were analyzed, and the percentage of diploid (2c), polyploid cells (>4c), and aneuploid cells was calculated using CytExpert software (Beckman Coulter).

### Hepatocyte nuclear ploidy analysis

Hepatocyte nuclear size quantitation was carried out on the basis of published methods (*37*) with some modifications. Briefly, diploid (2c) and tetraploid (4c) hepatocyte nuclear size (cells with round or oval nuclei) was estimated by quantitating the average area of ~1000 cells in H&E-stained liver sections taken from control 2- to 3-month-old C57BL/6J WT mice (*n* = 3), using ImageJ “Particle Analysis.” The area of 2c cells averaged 33μm^2^ (range, 22 to 38 μm^2^), and the area of 4c cells averaged 53 μm^2^ (range, 40 to 65 μm^2^). These ranges were then used to help define hyperpolyploid nuclear sizes (8c, 16c, 32c, and 64c), as per published methods (*37*). Annotation and counting of cells with diploid and polyploid content were carried out in NDP.view2, and percent cells were calculated from approximately 1000 hepatocytes. The number of cells with aberrant mitotic features and nuclear inclusions was estimated from the same images. At least 8 to 10 different FOVs (×40 magnification images) were selected randomly from each mouse liver, ensuring that central vein–portal vein axes were analyzed equally for each mouse. Data were obtained from 7 to 10 mice per genotype per group. Mono- and binucleated hepatocytes were manually distinguished from the H&E-stained tissues.

### Liver proteomic analysis

Global proteomic analyses of liver tissue from 3-, 12-, and >18-month-old (nontumor) male *Casp2^+/+^*, *Casp2^−/−^*, and *Casp2^C320S^* mice (*n* = 6 per genotype per age group). Briefly, 30 to 60 mg of liver tissue was homogenized in 300 to 600 ml of RIPA lysis buffer containing 2% SDS and supplemented with 1× Halt Protease and Phosphatase Inhibitor Cocktail, EDTA (Thermo Fisher Scientific). Tissue lysates were sonicated and cleared by centrifugation at 20,000*g* (4°C), and protein concentration was determined with BCA assay (Bio-Rad). Cell lysates (~20 μg of protein per replicate) were transferred to 0.5 ml of LoBind deep-well plate (Eppendorf) prepared for mass spectrometry (MS) analysis using the following modified SP3 protocol (*56*): Samples were subjected to simultaneous reduction and alkylation with a final concentration of 10 mM tris (2-carboxyethyl)phosphine and 40 mM 2-chloracetamide, followed by heating at 95°C for 10 min. Prewashed magnetic PureCube Carboxy agarose beads (20 μl; Cube Biotech) were added to all samples along with acetonitrile [ACN; 70% (v/v) final concentration] and incubated at room temperature for 20 min. Samples were placed on a magnetic rack, supernatants were discarded, and beads were washed twice with 70% ethanol and once with neat ACN. ACN was evaporated using a CentriVap (Labconco) before addition of digestion buffer [50 mM tris-HCl (pH 8)] containing 1 μg each of Lys-C (129-02541, Wako) and SOLu-Trypsin (EMS0004, Sigma-Aldrich). Trypsin/Lys-C on-bead digestion was performed with agitation (400 rpm) for 1 hour at 37°C, and samples were transferred to preequilibrated C18 StageTips (2× plugs of 3 M Empore resin, no. 2215) for cleanup. The eluates were lyophilized before being reconstituted in 0.1% formic acid/2% ACN for MS analysis.

Liquid chromatography (LC)–MS/MS analysis was performed on a timsTOF Pro MS (Bruker) with a CaptiveSpray source. Peptides were separated on a C_18_ fused silica column (inner diameter, 75 μm; OD, 360 μm by 15 cm in length, 1.6-μm C_18_ beads) packed into an emitter tip (Aurora, IonOpticks) using a custom nanoflow high-performance LC system (Thermo Fisher Scientific UltiMate 300 RSLCnano LC and PAL Systems CTC autosampler). Peptides were loaded onto the column at a constant flow rate of 600 nl/min with buffer A (99.9% Milli-Q water and 0.1% FA) and eluted with a 30-min linear gradient at 400 nl/min from 2 to 34% buffer B (90% ACN and 0.1% FA). The MS was operated in diaPASEF mode using Compass Hystar 5.1. The settings on the thermal ionization mass spectrometry analyzer were as follows: lock duty cycle to 100% with equal accumulation and ramp times of 100 ms; 1/K0, start: 0.6 V·s/cm^2^ and end: 1.6 V·s/cm^2^; capillary voltage, 1400 V; dry gas, 3 liters/min; dry temperature, 180°C. The data-independent acquisition (DIA) methods were set up using timsTOF control (v2.0.18.0) for two windows in each diaPASEF scan, with window placement overlapping the diagonal scan line for doubly and triply charged peptides in the mass/charge ratio (*m*/*z*)—ion mobility plane across 16 × 25 *m*/*z* precursor isolation windows (resulting in 32 windows) defined from *m*/*z* 400 to 1200, with 1-Da overlap, and CID collision energy ramped stepwise from 20 eV at 0.8 V·s/cm^2^ to 59 eV at 1.3 V·s/cm^2^. DIA-NN 1.8 was used for searching of timsTOF diaPASEF .d files in library-free mode with match between runs enabled. Data were searched against sequences from the *Mus musculus* proteome downloaded from UniProt (October 2021). The search was set to trypsin specificity, a peptide length of 7 to 30 residues, cysteine carbidomethylation as fixed, and the maximum number of missed cleavages at 2. Variable modifications included N-terminal acetylation and methionine oxidation and maximum variable modifications set to 1. Mass accuracy was set to 10 parts per million for both MS1 and MS2 spectra and the quantification strategy to robust LC (high precision).

Peptides identified in at least 50% in at least one group were analyzed with a total of 7140 proteins included in subsequent analyses. Missing values were imputed by applying Barycenter approach (v2-MNAR) implemented in msImpute (v.1.7.0). Protein log intensities were normalized using RUVIII (v.1.0.19). The probability of differential protein expression between groups was calculated using Limma (v.3.52.4), with paired-test pairwise comparisons and analysis of variance (ANOVA). A protein was determined to be significantly differentially expressed if the false discovery rate was ≤0.05. The MS proteomics datasets have been deposited to the ProteomeXchange Consortium and are available via the PRIDE database repository with the dataset identifier PXD063938 (www.ebi.ac.uk/pride/archive/projects/PXD063938).

### Enrichment analysis of biological processes and gene expression analyses

GSEA of pathways and genes was performed using the ReactomePA package (v.1.48.0). Venn diagrams were generated using BioVenn (*57*), and heatmaps were generated using Heatmapper (*58*). Analyses of gene alterations in HCC and HBC were done using BioPortal (www.cbioportal.org/). Correlation analysis between the CIN70 gene signature (*46*) and the ppaLD-13 gene signature (described here) in liver hepatocellular carcinoma [LIHC, The Cancer Genome Atlas (TCGA)] was carried out using GEPIA2 (http://gepia2.cancer-pku.cn) (*59*). Gene expression analyses for the 13 ppaLD proteins in MASLD and HCC were carried out in GepLiver (www.gepliver.org/). Survival analyses associated with genes in liver cancer (LIHC) were carried out using Kaplan-Meier Plotter (https://kmplot.com/analysis/). To quantify the daHep gene signature score, we first manually curated two gene sets representing up-regulated and down-regulated daHep-associated genes (*47*) based on differential expression analysis with an adjusted *P* < 0.05 and absolute log_2_ fold change > 0.6. We then applied the hacksig R package using the single-sample GSEA (ssGSEA) approach to computing enrichment scores for each sample (*60*). The analysis was performed using normalized protein expression data as input, with the following parameters: method = “ssgsea,” sample_norm = “all,” and rank_norm = “rank.” The resulting enrichment scores were expressed as fold changes relative to WT samples. Statistical significance between groups was assessed using Wilcoxon rank-sum tests (nonparametric).

### Statistical analysis of data

GraphPad Prism software (v10.5.0, GraphPad Inc.) and Microsoft Excel (v16.99.1) was used for generating graphs and for statistical analyses. Survival curves were generated using Kaplan-Meier method, and Fisher’s exact test was used to analyze survival data. Statistical analysis was performed using chi-squared test (*χ*^2^), two-tailed unpaired t-test, nonparametric *t* test, or parametric *t* test with Welch’s correction, unless otherwise described. Data are expressed as means ± SEM or means ± SD, with *P* < 0.05 considered significant.

## Supplementary Materials

### The PDF file includes:

Supplementary Methods Figs. S1 to S8 Legends for datasets S1 to S4

### Other Supplementary Material for this manuscript includes the following:

Datasets S1 to S4
