# Supplementary material for: Caspase-2 deficiency drives pathogenic liver polyploidy and increases age-associated hepatocellular carcinoma in mice
Source: Sci Adv. 2026 Jan 1;12(1):eaeb2571. doi: 10.1126/sciadv.aeb2571 (PMC12757054; doi:10.1126/sciadv.aeb2571)
Supplement: Supplementary file 1 — Supplementary Methods Figs. S1 to S8 Legends for datasets S1 to S4 [file sciadv.aeb2571_sm.pdf]

Science Advances
AAAS

Supplementary Materials for

Caspase-2 deficiency drives pathogenic liver polyploidy and increases age-associated hepatocellular carcinoma in mice

Loretta Dorstyn *et al.*

Corresponding author: Loretta Dorstyn, loretta.dorstyn@adelaide.edu.au; Sharad Kumar, sharad.kumar@adelaide.edu.au

Sci. Adv. **12**, eaeb2571 (2026)
DOI: 10.1126/sciadv.aeb2571

The PDF file includes:

Supplementary Methods
Figs. S1 to S8
Legends for datasets S1 to S4

Other Supplementary Material for this manuscript includes the following:

Datasets S1 to S4

Supplementary Text

Supplementary Methods

Histological tissue examination

Adipocyte size measurements: Images of H&E stained gWAT sections from old mice (18-24 month-old) were taken at 10X magnification and analysed using Adiposoft plugin in ImageJ. Briefly, the scale bar and threshold were adjusted and set for each image before Adiposoft analysis. All cells "on-edges" were excluded and cells manually deleted or added where incompletely marked. Average adipocyte size was determined from at least 1000 cells per gWAT, for 6-7 female and 6-7 male mice per genotype.

Skin thickness quantitation: Skin layers were measured as previously described [9] from H&E-stained skin sections. Briefly, epidermal thickness was measured from stratum basale to stratum granulosum (excluding stratum corneum); dermal thickness was the distance between the epidermis; hypodermis (dermal white adipose tissue, dWAT) layer thickness was measured as the distance between the dermis and the muscle layer (panniculus carnosus). Approximately 15-20 measurements of each layer were taken at randomly selected areas, and the average thickness from n= 5-6 sections per genotype was calculated.

Mouse Plasma Biochemical Profiling

Total cholesterol was determined in plasma by automated analysis (SA Pathology, Adelaide, Australia). Blood glucose was determined using an automated monitor (Accu-Chek, Roche).

Cytokine Array

Levels of chemokine and cytokines were examined using a Proteome Profiler Mouse Cytokine Array Kit, Panel A (R&D systems) as per manufacturer's instructions using 50-100 µl plasma each from n=3 mice per genotype and overnight incubation at 4 °C on a rocking platform shaker. Following a series of washes, blots were incubated with diluted Streptavidin-HRP or with Streptavidin-AP (1:2000 in Array Buffer 6) for 1 h at room temperature on a rocking platform shaker and signals detected by ECL with Chemi Reagent Mix on a Fuji LAS4000 System (GE Healthcare) or ECF using AttoPhos reagent (Promega) and imaged on a Typhoon FLA 9000 (GE Healthcare).

Ferroptosis Assays

The Huh7 hepatoma cell line was maintained in a humidified incubator at 37 °C with 10% CO_2 in high-glucose Dulbecco's Modified Eagles Medium, DMEM (DMEM, Sigma-Aldrich) supplemented with 10% foetal bovine serum (JRH Biosciences, Lenexa, KS, USA), 0.2 mM l-glutamine (Sigma-Aldrich), 15 mM HEPES (Sigma-Aldrich) and 100 µM penicillin/streptomycin (Sigma-Aldrich).

Cells were transfected with 40 nM *Casp2 siRNA* as previously described (*14*) using Lipofectamine RNAiMAX transfection reagent (Life Technologies) as per the manufacturer's instructions. For MTS assay, after 24h, cells were re-seeded into a 96 well plate (Falcon®), at 3500 cells per well in 100 µl culture media. The next day culture medium (100 µl) containing DMSO, 10 µM erastin, or 500 nM RSL3 was added, followed by 24 h incubation at 37 °C, 10%

CO_2. Twenty-five microliters of CellTiter 96® AQueous One Solution Cell Proliferation Assay (Promega) were added to each well, followed by incubation for 3 h in a 10% CO2 incubator at 37 °C. Absorbance was measured at 490 nm using a FLUOstar Omega (BMG Labtech, Ortenberg, Germany). Control wells (culture media only) were used to detect the cell-free background absorbance.

For protein extraction, cells were transfected with siRNAs in D60 cell culture dishes. Following 16-24 h, the culture medium was replaced with media containing 10 μM erastin 500 nM RSL3, with or without the ferroptosis inhibitors ferrostatin-1 (20 μM). Cells were treated with ferroptosis drugs for 24 hours and then protein lysates were prepared from cells in RIPA buffer (25 mM Tris/HCl pH 7.4, 150 mM NaCl, 1% nonyl-phenoxylpolyethoxylethanol, 1% sodium deoxycholate, 0.1% sodium dodecyl sulfate) in the presence of protease/phosphatase inhibitor cocktail (Thermo Scientific, Rockford, Illinois, USA).

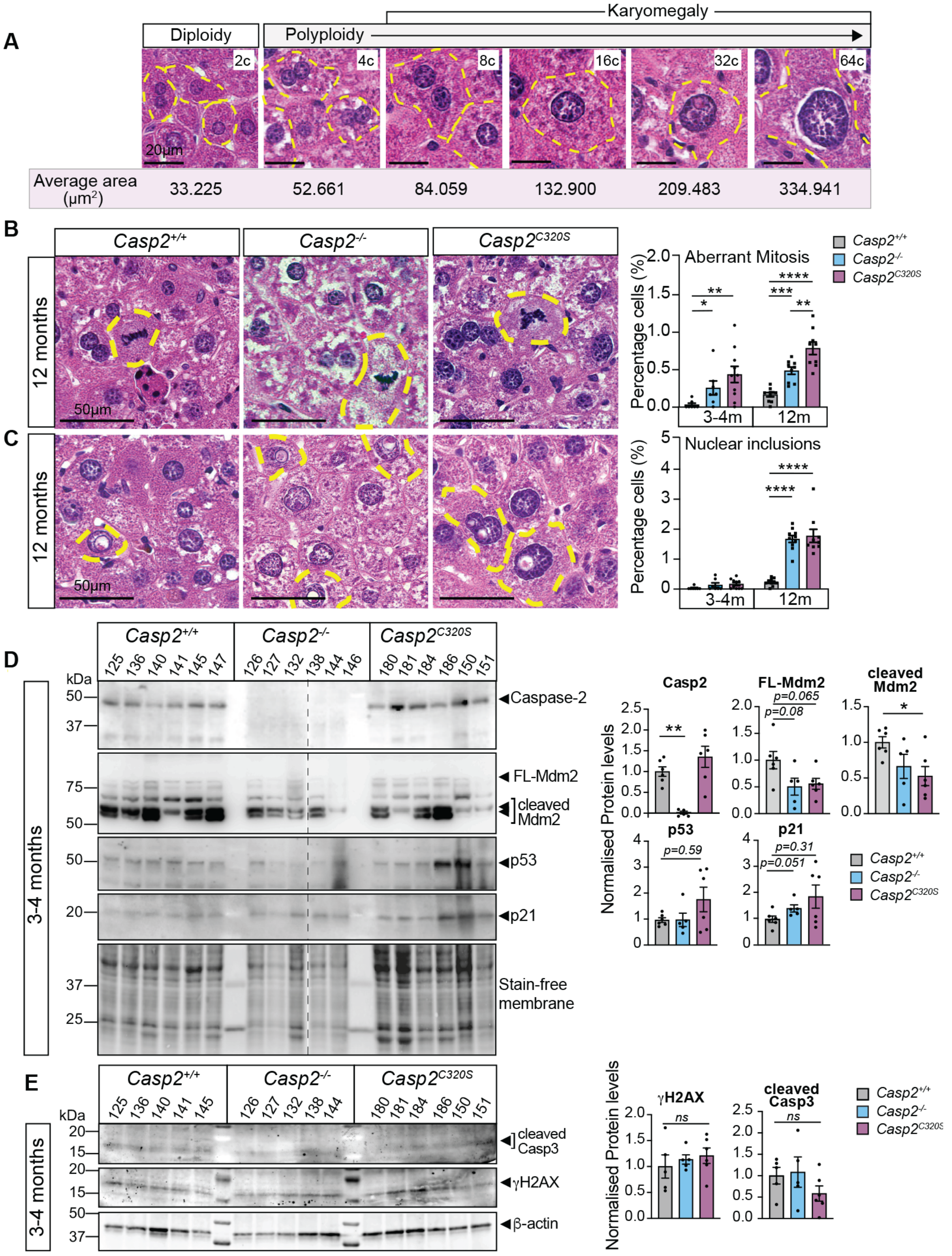

Fig. S1. Hepatocyte ploidy quantitation and mitotic catastrophe signaling in Casp2 deficient mouse livers.

(A) Representative images showing the nuclear morphology and nuclear size associated with ploidy content of hepatocytes. The average area of hepatocyte nuclei is indicated and determined based on published calculations for the relationship between the cross-sectional area of the spherical nucleus and the DNA content (2c-64c) (50). Individual cells with the indicated ploidy content are encircled (yellow). **(B-C)** Representative images of H&E-stained liver sections highlighting mitotic cells **(B)** and nuclear inclusions **(C).** Quantitation of the percentage cells with aberrant mitoses and nuclear inclusions calculated in H&E-stained liver sections from 3-4-

month-old and 12-month-old *Casp2*$^{+/+}$, *Casp2*$^{-/-}$ and *Casp2*^{C320S} mice. For all graphs, values are mean ± SEM, from 8-10 random fields of view and n=4-5 independent liver samples per genotype for each age cohort. Significance is indicated as *p<0.05, **p<0.01, ***p<0.001, ****p<0.0001. **(D)** Representative immunoblots of liver lysates showing Mdm2 cleavage and levels of caspase-2, p53, p21. Stain-free membrane indicates protein loading. **(E)** Representative immunoblots of liver lysates showing cleaved caspase-3 and γH2AX. n=6 independent liver samples per genotype are shown. β-actin levels indicate protein loading. In **(D)** and **(E)**, protein expression levels are quantitated and shown as fold change from expression in *Casp2*$^{+/+}$ liver lysates, relative to protein loading levels. Dotted line indicates where membrane was cut and joined together. Values are mean ± SEM. Significance is indicated as *p<0.05, **p<0.01, *ns*= not significant.

Supplementary Figure S2

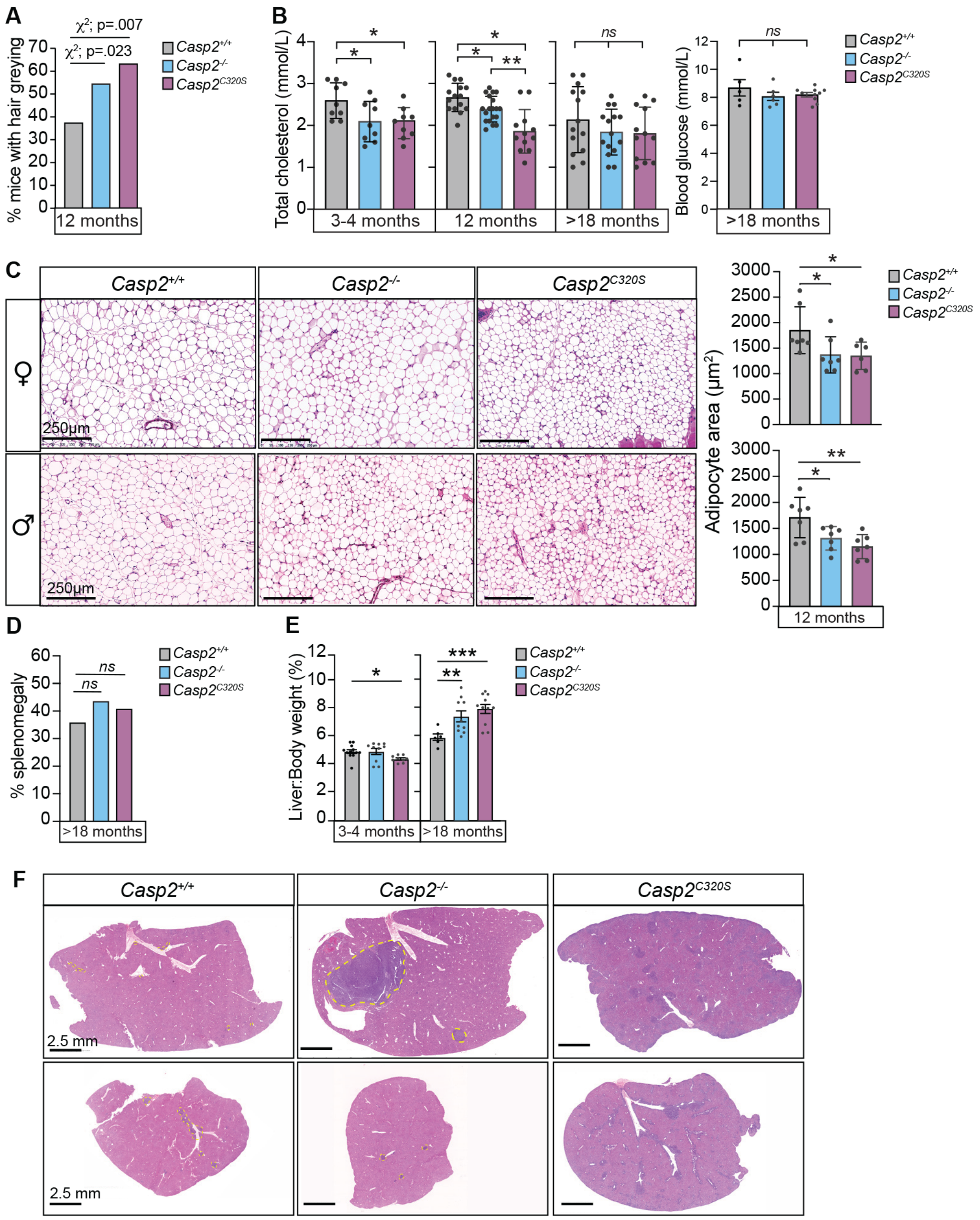

Fig. S2. Ageing phenotypes in *$Casp2^{C320S}$* mice.

(A) Incidence of hair greying in 12-month-old male *$Casp2^{+/+}$*, *$Casp2^{-/-}$* and *$Casp2^{C320S}$* mice. Significance was determined by Chi-squared test: [*$Casp2^{+/+}$* v *$Casp2^{-/-}$* :$\chi^2(1, N = 15) = 5.153$, $p = .023$] and [*$Casp2^{+/+}$* v *$Casp2^{C320S}$*: $\chi^2(1, N = 15, 20) = 11.52$, $p = .007$]. Values represent mean percentage (%) mice (n=12-15 per genotype). **(B)** Total cholesterol determined in plasma samples from *$Casp2^{+/+}$*, *$Casp2^{-/-}$* and *$Casp2^{C320S}$* mice at different age cohorts: Values represent mean percentage (%) mice at 3–4-month-old (n=6-8), 12-month-old (n=6-8) and >18-month-old (n=13-16). Blood glucose levels were determined in 12–24-month-old mice (n=6-10). Values

represent mean percentage (%) mice with significance indicated as *p<0.05, **p<0.01, *ns*= not significant. **(C)** Histological analyses of gWAT from old (20-24-month-old) female and male *Casp2*$^{+/+}$, *Casp2*$^{-/-}$ and *Casp2*^{C320S} mice. Scale= 250µm. Bar graphs show mean adipocyte size ± SEM, calculated from >500 cells from 5 random fields of view (n=4-6). Significance is indicated as *p<0.05, **p<0.01. **(D)** Frequency of splenomegaly in >18-month-old *Casp2*$^{+/+}$, *Casp2*$^{-/-}$ and *Casp2*^{C320S} mice. (n=14-28). *ns*=not significant as determined by Chi-squared test statistics. **(E)** Liver:Body weight ratios calculated in young (3–4-month-old, n=6-8) and old (12-24-month-old, n=13-16) *Casp2*$^{+/+}$, *Casp2*$^{-/-}$ and *Casp2*^{C320S} mice. Values are mean ± SEM. Significance is indicated as *p<0.05, **p<0.01, ***p<0.001. **(F)** Representative images of liver sections stained with H&E showing heterogeneous lymphoma phenotypes with larger distinct areas of peri-portal lymphomatous infiltration (bordered in yellow) and more severe wide-spread lymphoma infiltration in livers from *Casp2*^{C320S} mice.

Supplementary Figure S3

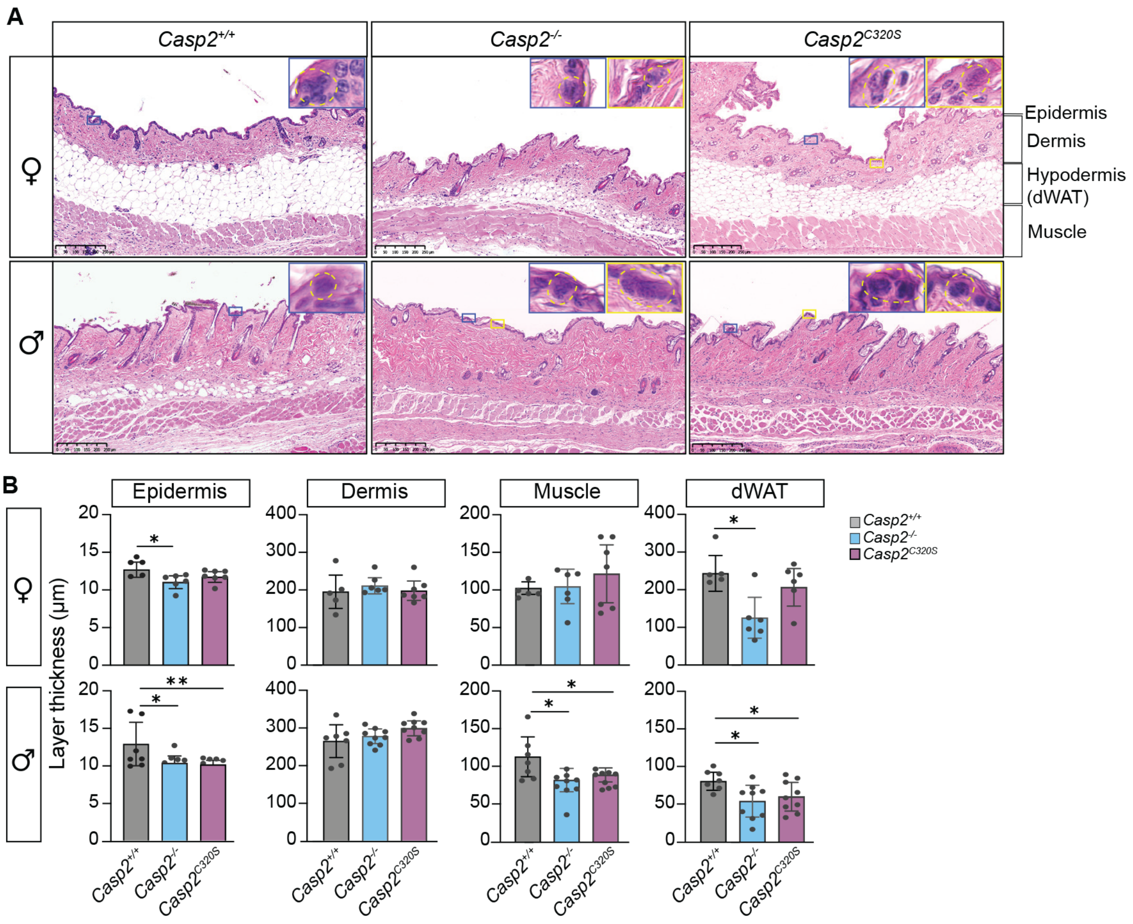

Fig. S3. Histological analysis of skin from *Casp2*^{C320S} mice.

(A) Representative images of Haematoxylin and Eosin-stained skin sections from 24-month-old female and male, *Casp2*$^{+/+}$, *Casp2*$^{-/-}$ and *Casp2*^{C320S} mice. Scale=250μm. Insets are higher magnification of epidermal regions showing cells with enlarged or multiple nuclei. **(B)** Quantification of layer thickness (μm) for epidermis, dermis, hypodermis (dWAT) and muscle layers from 24-month-old female and male mice. Average thickness of each layer was determined from 8-10 measurements in each of 5 random fields of view across 1-2cm skin. Values are mean ± SEM, n=5-6 sections per genotype. Significance is indicated as *$p<0.05$, **$p<0.01$.

Supplementary Figure 4

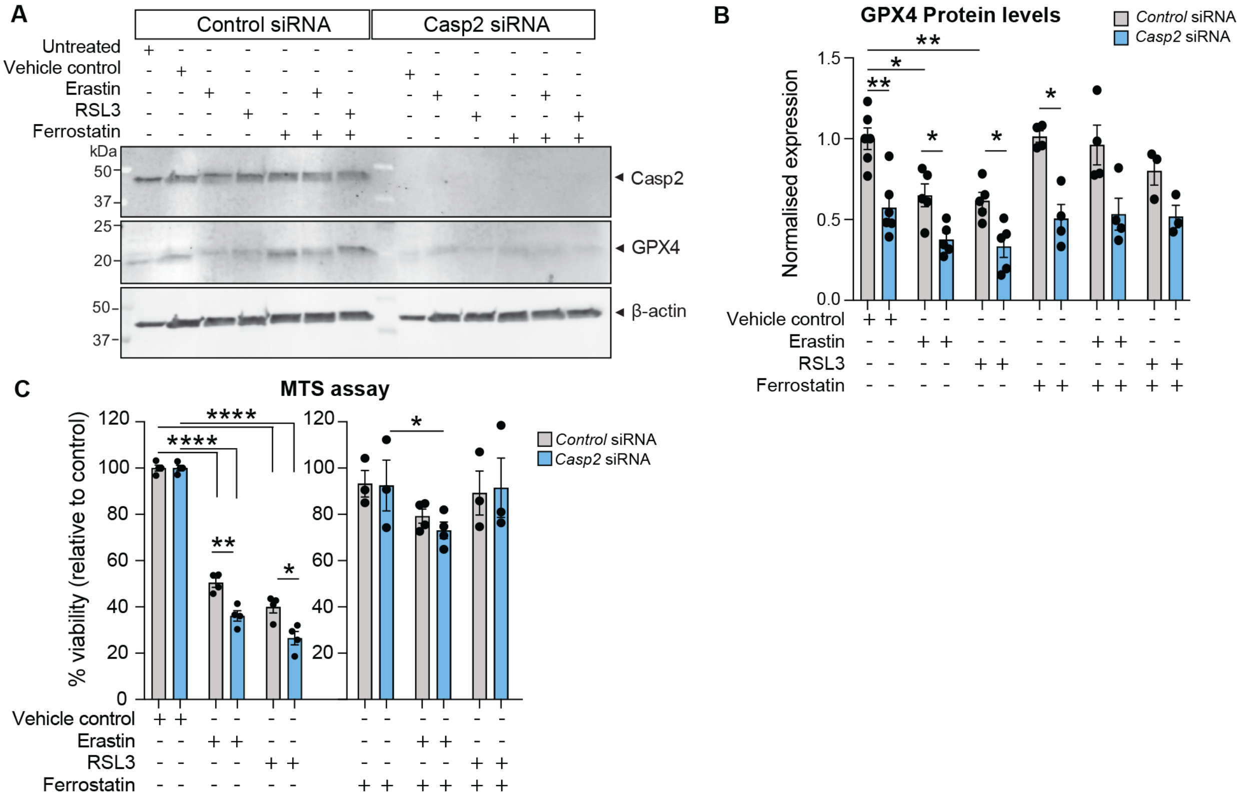

Figure S4. Casp2 depletion increases ferroptosis in Huh7 cells.

(A) Immunoblot analysis of caspase-2 and GPX4 expression in Huh7 cells with control or *CASP2* siRNA, followed by treatment with DMSO (vehicle control) or ferroptosis inducing drugs, erastin (10 μM) or RSL3 (500 nm), with or without ferrostatin (20 μM) for 24 h. β-actin is shown as the loading control. **(B)** Quantitation of GPX4 protein levels from **(A)** are represented as fold change from control siRNA vehicle control samples or from control siRNA ferrostatin treated cells (n=3-4 independent experiments). Values are mean ± SEM. Significance is indicated as *$p<0.05$, **$p<0.01$. **(C)** Viability of Huh7 cells with control or *CASP2* siRNA, treated with ferroptosis drugs as in **(A)** at 24 h post-treatment. n=3-4 independent experiments. Values are mean ± SEM. Significance is indicated as *$p<0.05$, **$p<0.01$, ****$p<0.0001$.

Supplementary Figure S5

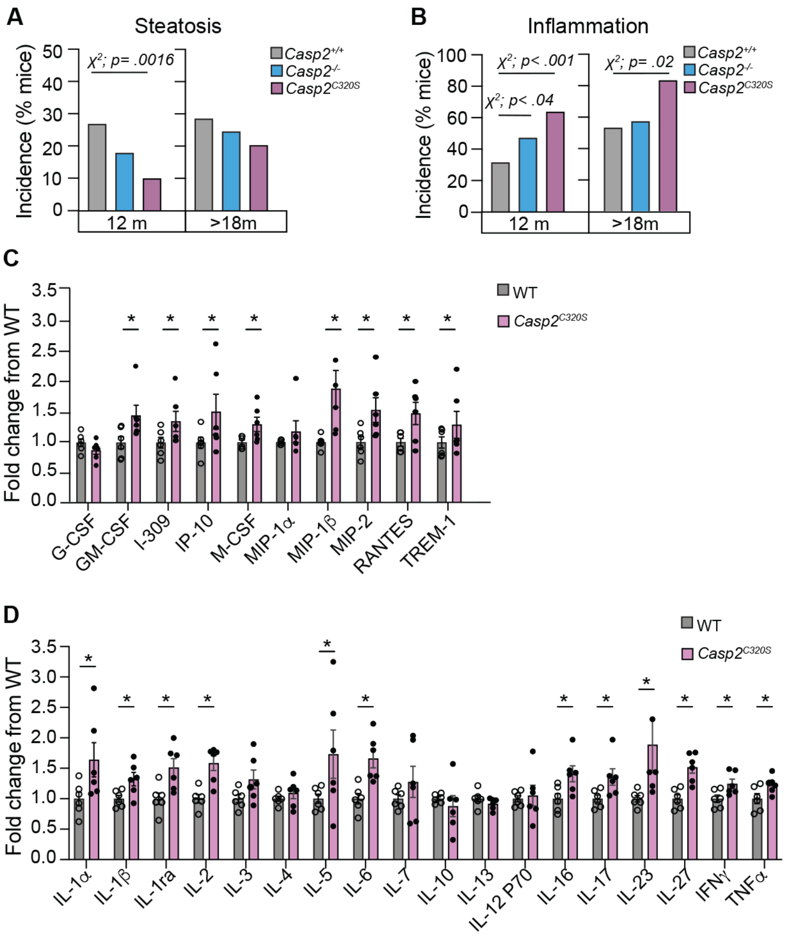

Fig S5. Liver steatosis and increased inflammation in aged Casp2- deficient mice. **(A-B)** Incidence of **(A)** steatosis and **(B)** inflammation in 12-month-old and in >18-month-old male *Casp2*$^{+/+}$, *Casp2*$^{-/-}$ and *Casp2*^{C320S} mice. Significance was determined by Chi-squared test as indicated. **(C-D)** Increased plasma inflammatory mediators associated with liver disease, from 12-month-old *Casp2*$^{+/+}$ and *Casp2*^{C320S} mice, including **(C)** chemokines and **(D)** cytokines, determined using a Cytokine profile array. Values are mean ± SEM; n= 5-6 independent plasma samples per genotype. Fold change of dot blot signal intensity was calculated in Image J. Significance is indicated as *p<0.05.

Supplementary Figure S6

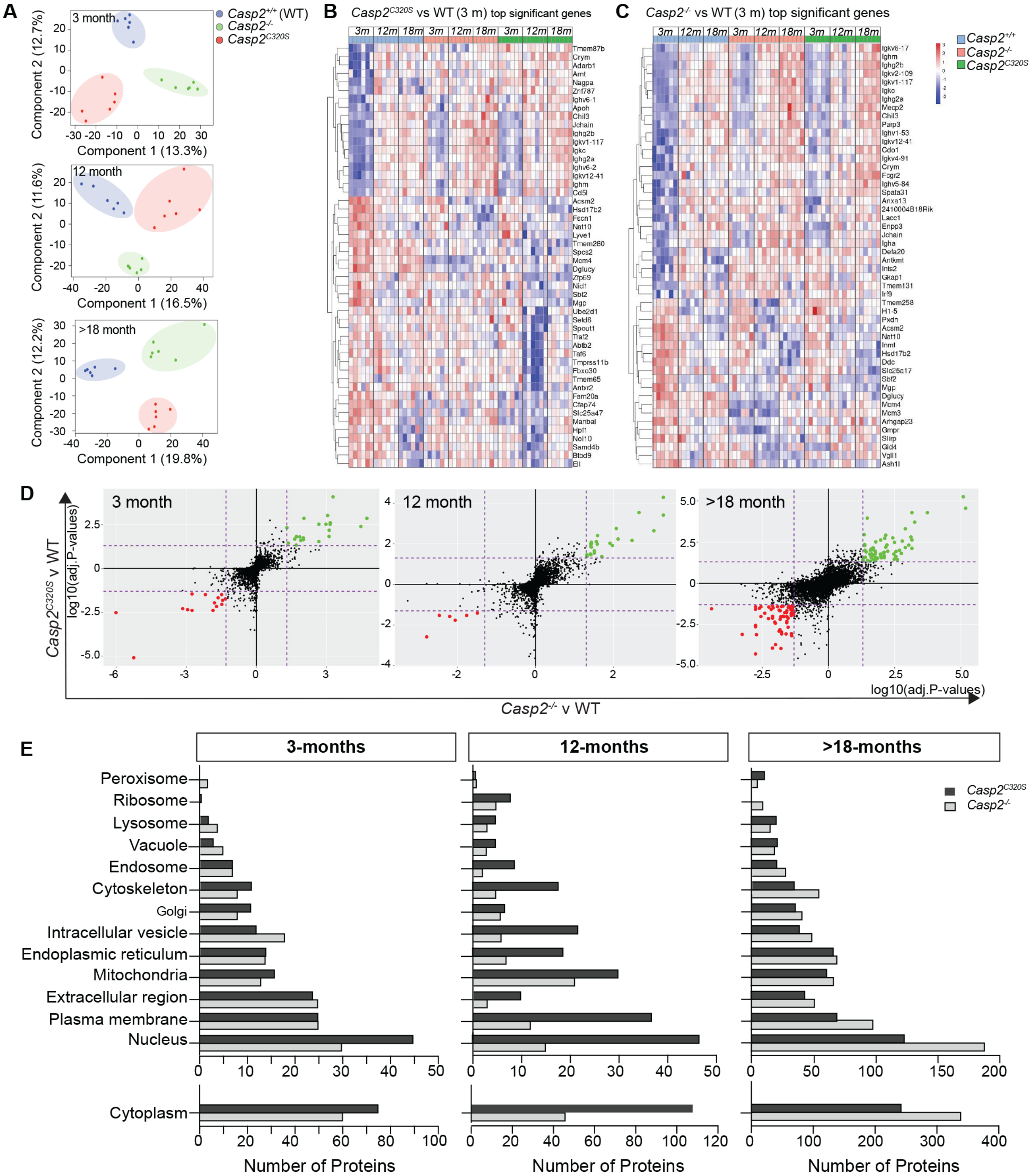

Figure S6. Age related liver proteomic comparisons from Casp2-deficient mice. (A) PCA plots of liver proteomes showing distinct clustering of *Casp2*$^{+/+}$ (n=6), *Casp2*$^{-/-}$ and (n=6) *Casp2*^{C320S} (n=6) samples from each age cohort. **(B-C)** Heat maps of differentially abundant proteins in 3-month-old livers comparing *Casp2*^{C320S} v *Casp2*$^{+/+}$ and *Casp2*$^{-/-}$ v *Casp2*$^{+/+}$. **(D)** Log-log plots displaying the proportion of differentially abundant proteins that are either increased (red) or decreased (blue) when comparing *Casp2*^{C320S} v *Casp2*$^{+/+}$ and *Casp2*$^{-/-}$ v

Casp2$^{+/+}$ liver proteomes. **(E)** Subcellular localisation analysis of differentially abundant proteins from *Casp2*^{C320S} and *Casp2*$^{-/-}$ v *Casp2*$^{+/+}$ liver proteomes at each age cohort.

Supplementary Figure S7

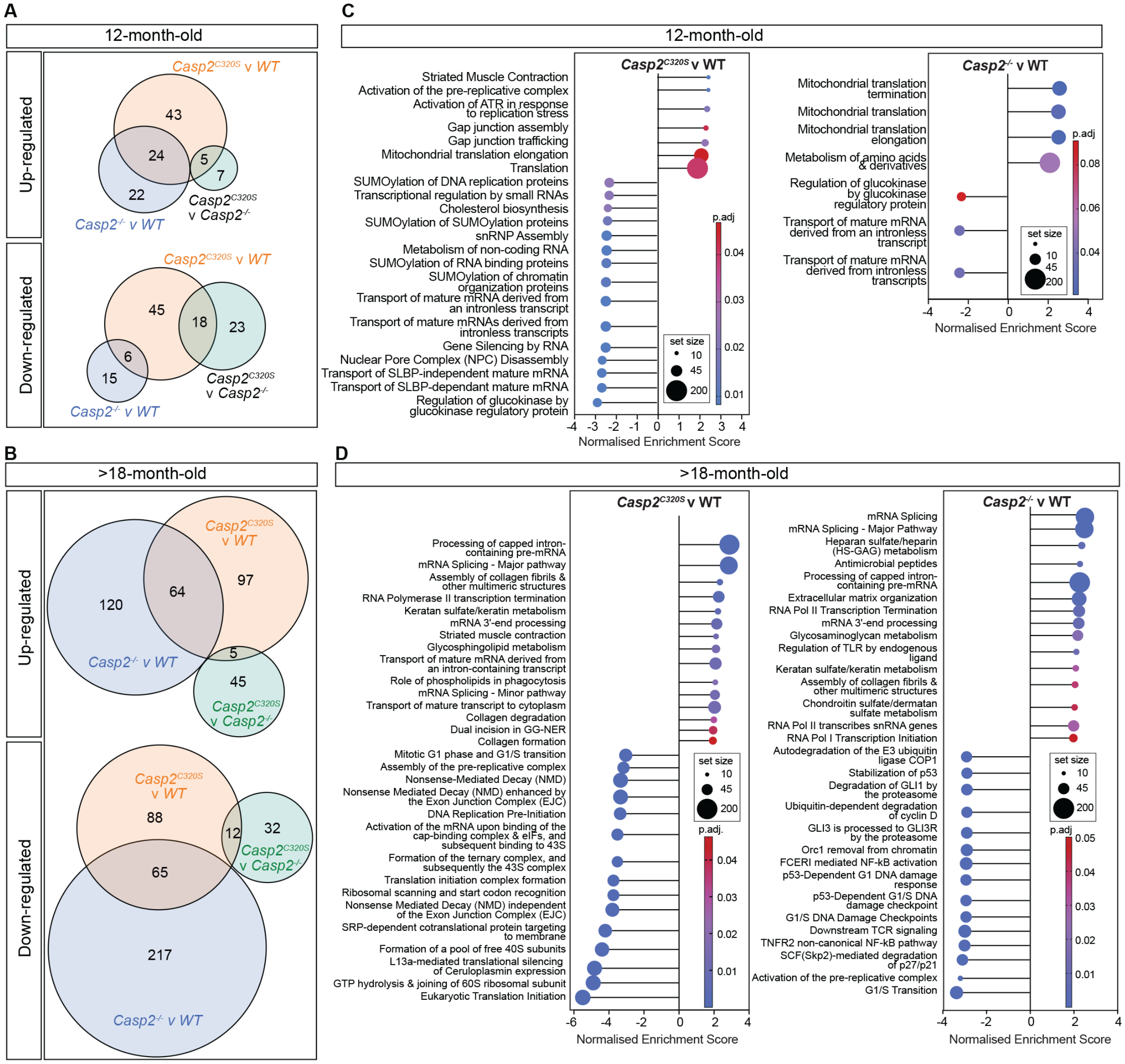

Figure S7. Comparisons and GSEA of differentially abundant protein detected in 12-month and >18-month-old livers from Casp2-deficient mice.
(A-B) Venn diagram summary of unique and overlapping proteins that are significantly upregulated or down-regulated in the indicated comparison groups, from **(A)** 12-month-old and from **(B)** >18-month-old liver samples. **(C-D)** Gene set enrichment analyses (GSEA) illustrating the top 15 altered pathways associated with differentially abundant proteins in *Casp2*^{C320S} v *Casp2*$^{+/+}$ and *Casp2*$^{-/-}$ v *Casp2*$^{+/+}$ liver proteomes from the indicated age cohorts.

Supplementary Figure S8

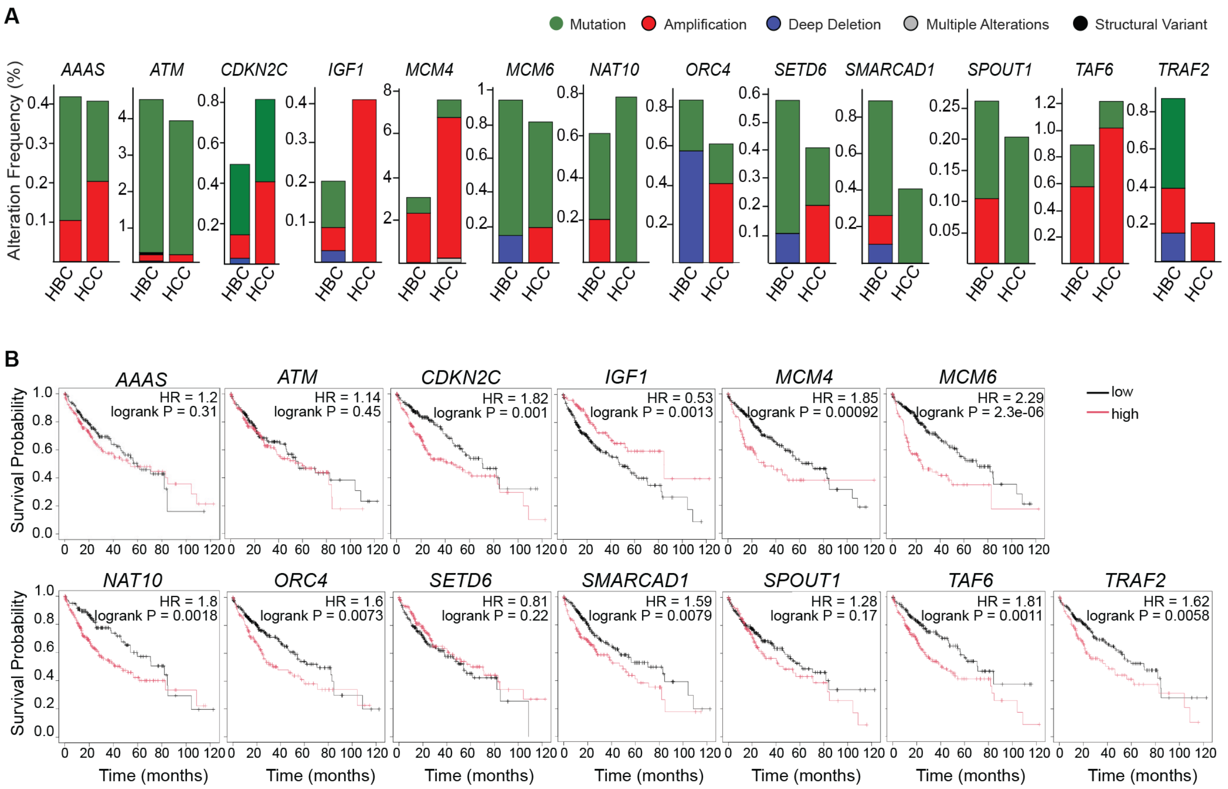

Figure S8. Association of the ppaLD protein signature with gene alterations and survival in liver cancer.

(A) Gene alteration frequency for the indicated ppaLD genes in human hepatocellular carcinoma (HCC) and hepatobiliary cancer (HBC), determined using cBioPortal for Cancer Genomics v6.3.3 (https://www.cbioportal.org/). **(B)** Kaplan–Meier plots showing the association of the expression of the indicated ppaLD signature gene, with patient survival in liver cancer (LIHC, TCGA).

Data Table S1 (i-vi) (separate file)

Lists of differentially abundant proteins in *Casp2^{C320S}* and *Casp2$^{-/-}$* livers compared to WT (*Casp2$^{+/+}$*) in each age cohort. (i) *Casp2^{C320S}* v *Casp2$^{+/+}$* (3m) (ii) *Casp2$^{-/-}$ v Casp2$^{+/+}$* (3m). (iii) *Casp2^{C320S}* v *Casp2$^{+/+}$* (12m) (iv) *Casp2$^{-/-}$ v Casp2$^{+/+}$* (12m). (v) *Casp2^{C320S}* v *Casp2$^{+/+}$* (>18m) (vi) *Casp2$^{-/-}$ v Casp2$^{+/+}$* (>18m). Upregulated and downregulated proteins are indicated. Corrected LogFC= log2-fold change of expression between genotypes examined, with adjusted *p* values are indicated.

DataTable S2 (i-iii) (separate file).

Protein lists associated with Venn Diagram protein comparisons between *Casp2^{C320S}* v *Casp2$^{+/+}$*, *Casp2$^{-/-}$ v Casp2$^{+/+}$* and *Casp2^{C320S}* v *Casp2$^{-/-}$* liver proteomes, for each age-cohort. (i) 3-months (ii) 12 months and (iii) >18-month-old livers. Lists are associated with Figure 8A and Extended Data Figure 7.

Data Table S3. (i-vi) (separate file).

Gene Set Enrichment Analyses (GSEA) of significantly altered Reactome pathways in *Casp2^{C320S}* or in *Casp2$^{-/-}$* livers compared to the *Casp2$^{+/+}$* liver proteome for each age-cohort. (i) *Casp2^{C320S}* v *Casp2$^{+/+}$* (3m) (ii) *Casp2$^{-/-}$ v Casp2$^{+/+}$* (3m). (iii) *Casp2^{C320S}* v *Casp2$^{+/+}$* (12m) (iv) *Casp2$^{-/-}$ v Casp2$^{+/+}$* (12m). (v) *Casp2^{C320S}* v *Casp2$^{+/+}$* (>18m) (vi) *Casp2$^{-/-}$ v Casp2$^{+/+}$* (>18m). Normalized enrichment scores (NES), Set size, adjusted pvalues and genes associated with enriched pathways are listed.

Data Table S4. (i-iii) (separate file).

Comparative analysis between the daHep signature and differentially abundant proteins in *Casp2^{C320S}* or in *Casp2$^{-/-}$* livers in each age- group. (i) Protein lists associated with Venn Diagram protein comparisons with the daHep gene signature for each age-cohort. Lists are associated with Figure 8A. (ii) daHep gene signature score analysis for differentially abundant proteins identified in *Casp2^{C320S}* or in *Casp2$^{-/-}$* liver samples from all age groups. The daHep score was calculated independently for upregulated genes (up) and down-regulated genes (down) with log2FC≥0.6 and adjusted p value<0.05.
